# Structure and dynamics of a constitutively active neurotensin receptor

**DOI:** 10.1038/srep38564

**Published:** 2016-12-07

**Authors:** Brian E. Krumm, Sangbae Lee, Supriyo Bhattacharya, Istvan Botos, Courtney F. White, Haijuan Du, Nagarajan Vaidehi, Reinhard Grisshammer

**Affiliations:** 1 Membrane Protein Structure Function Unit, National Institute of Neurological Disorders and Stroke, National Institutes of Health, Department of Health and Human Services, Rockville, Maryland 20852, United States; 2 Department of Molecular Immunology, Beckman Research Institute of the City of Hope, 1500 E Duarte Road, Duarte, California 91010, United States; 3 Laboratory of Molecular Biology, National Institute of Diabetes and Digestive and Kidney Diseases, National Institutes of Health, Department of Health and Human Services, Bethesda, Maryland 20892, United States

## Abstract

Many G protein-coupled receptors show constitutive activity, resulting in the production of a second messenger in the absence of an agonist; and naturally occurring constitutively active mutations in receptors have been implicated in diseases. To gain insight into mechanistic aspects of constitutive activity, we report here the 3.3 Å crystal structure of a constitutively active, agonist-bound neurotensin receptor (NTSR1) and molecular dynamics simulations of agonist-occupied and ligand-free receptor. Comparison with the structure of a NTSR1 variant that has little constitutive activity reveals uncoupling of the ligand-binding domain from conserved connector residues, that effect conformational changes during GPCR activation. Furthermore, molecular dynamics simulations show strong contacts between connector residue side chains and increased flexibility at the intracellular receptor face as features that coincide with robust signalling in cells. The loss of correlation between the binding pocket and conserved connector residues, combined with altered receptor dynamics, possibly explains the reduced neurotensin efficacy in the constitutively active NTSR1 and a facilitated initial engagement with G protein in the absence of agonist.

G protein-coupled receptors (GPCRs) are highly dynamic and versatile signalling molecules that mediate second messenger responses within the cell. Binding of an extracellular agonist causes conformational changes in the receptor, triggering activation of signalling partners such as G proteins or arrestin molecules on the intracellular side of the membrane. Advances in the structural biology of GPCRs have yielded high-resolution structural snapshots of inactive^1^, active-intermediate^2^,^3^ and active receptor conformations^4^,^5^,^6^,^7^,^8^,^9^,^10^ and have provided insight into the activation mechanism likely shared by class A GPCRs^4^,^5^,^7^,^9^,^10^,^11^. Subtle, agonist-specific changes in the receptor agonist-binding pocket lead to rearrangement in the packing of connector residues in the receptor centre, effecting larger conformational changes on the intracellular side of the GPCR such as the outward movement of transmembrane helix (TM) 6.

Constitutive (basal) activity is defined as ligand independent activity, resulting in the production of a second messenger in the absence of an agonist. Many wild-type GPCRs have been found to be constitutively active, but also mutations in any part of the receptor can cause constitutive activation (constitutively active mutants)^12^. The extent of the basal activity of GPCRs varies and the addition of an agonist may or may not increase the signalling response above the basal level. Constitutively active mutants have been linked to human disease^13^. For example, certain mutations in the thyrotropin receptor are associated with hyperthyroidism, mutations in the visual pigment rhodopsin cause night blindness and retinitis pigmentosa, and mutations in the parathyroid hormone receptor result in skeletal deformations. While the pharmacological and pathological properties of constitutively active receptors have been studied extensively^13^, the structural basis of constitutive activity remains largely unexplored.

Our research focuses on the neurotensin receptor 1 (NTSR1, ref. 14). Its endogenous agonist ligand neurotensin (NTS) is a 13-amino acid peptide that acts as a neurotransmitter and a hormone in peripheral tissues and the nervous system^15^. NTS displays a wide range of biological activities with important aspects in cancer cell growth and the pathogenesis of schizophrenia^16^,^17^. Previously, we reported the structure of a thermostable mutant, NTSR1-GW5 (ref. 3) bound to the C-terminal portion (NTS_8-13_) of neurotensin in an active-intermediate conformation with six thermostabilizing mutations providing insight into the binding mode of a peptide agonist. Agonist-occupied NTSR1-GW5 was unable to activate nucleotide exchange in G proteins, indicating that the mutations restricted NTSR1 to relate NTS binding to G protein activation. Subsequent analysis of the effect of the mutations resulted in the triple revertant NTSR1-ELF, which was able to stimulate nucleotide exchange at Gαq in response to NTS to almost wild-type level^18^.

Here we report the crystal structure of the constitutively active NTSR1-EL mutant in complex with NTS_8-13_ at 3.3 Å resolution, and the pharmacological and dynamic behaviour of the receptor. NTSR1-EL is an active-intermediate receptor with a sequence that is almost identical to NTSR1-ELF except that it contains the F358A mutation. NTSR1-EL displays pronounced constitutive activity, while NTSR1-ELF does not. As found with other constitutively active GPCRs, NTS-induced activation of G protein is reduced in NTSR1-EL, in contrast to NTSR1-ELF and the wild-type receptor (NTSR1-WT). Comparison of the crystal structures of NTSR1-EL and NTSR1-ELF^18^ and molecular dynamics (MD) simulations show that structural rearrangements within NTSR1-EL, combined with altered receptor dynamics, may facilitate the initial contact with G protein in the absence of agonist, while the loss of correlated coupling between the binding pocket and connector residues may explain the reduced effect of NTS on signalling.

## Results

We describe below the structure, pharmacology and molecular dynamics of the constitutively active mutant NTSR1-EL to answer two fundamental questions: What are the features of NTSR1-EL promoting constitutive activity; and why is NTS not effective in stimulating a signalling response to the level of the wild-type receptor.

Receptor signalling relies on the communication between the agonist-occupied ligand-binding pocket, the connector region, and the G protein-coupling interface of the receptor, linking relatively small conformational changes within the ligand-binding site and the connector region to large changes at the intracellular receptor surface. Although interconnected, the ligand-binding site and the G protein interface show only weak allosteric coupling^19^,^20^,^21^ and each of the three regions may switch individually between multiple distinct conformations^20^. We therefore discuss the structural rearrangements in these regions, but we do not provide an exhaustive correlation analysis of the ligand-binding pocket, connector and G protein-coupling interface because there are currently no crystal structures of NTSR1 in an antagonist-bound inactive-state nor a fully active G protein-bound state available for comparison, and the microsecond-scale MD simulations of NTSR1 do not allow observing the transition from active-intermediate to inactive or fully active conformations.

### NTSR1 construct nomenclature

The NTSR1 mutants discussed in this study are described in Supplementary Table 1. They are based on NTSR1-GW5, a receptor with six thermostabilizing mutations (A86L^1.54^, E166A^3.49^, G215A^ECL2^, L310A^6.37^, F358A^7.42^ and V360A^7.44^) (superscripts are the Ballesteros-Weinstein numbers^22^). Reverting three of these mutations resulted in NTSR1-ELF (with E166^3.49^, L310^6.37^, and F358^7.42^) (ref. 18). NTSR1-EL contains the wild-type residues E166^3.49^ and L310^6.37^, but also has the F358A^7.42^ mutation, in addition to A86L^1.54^, G215A^ECL2^, and V360A^7.44^ found in NTSR1-ELF. The W321A^6.48^ point mutation is indicated in the relevant constructs. One other mutant NTSR1 is discussed, NTSR1-TM86V, which was evolved to be more thermostable with neurotensin bound by Plückthun’s laboratory^23^. This mutant harbours 11 mutations and the structure has been determined^24^.

For the structure determination of NTSR1-EL-T4L, we obtained well-diffracting crystals using the chimeric T4 lysozyme (T4L, cysteine-free bacteriophage T4 lysozyme with C54T, C97A) approach^25^, where most of intracellular loop (ICL) 3 is replaced by T4L. For pharmacological analyses, the NTSR1 constructs contain the wild-type ICL3, not T4L. The identity of the relevant constructs is unambiguously specified in Figures and Tables.

All models derived from MD simulations use the respective mutant name, but have the ‘MD’ subscript to distinguish them from receptor models based on crystal structures or constructs used for pharmacological analyses (for example NTSR1-EL_MD_ versus NTSR1-EL). The term NTSR1_MD_ refers collectively to NTSR1-EL_MD_ and NTSR1-ELF_MD_.

The docking models NTSR1-SR_dock_ and NTSR1-NTS_dock_ are based on the crystal structure of active-intermediate NTSR1-LF^18^, modified with the wild-type NTSR1 sequence, and contain the antagonist SR48692 (ref. 26) or NTS_8-13_, respectively, in the ligand-binding pocket.

### NTSR1-EL is constitutively active

The current study characterizes the constitutive activity of NTSR1-EL towards G proteins; the analysis of arrestin recruitment by NTSR1-EL will be subject of a separate study.

**Figure 1:**
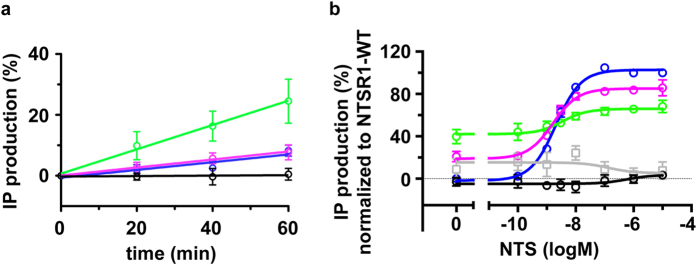
Constitutive activity of NTSR1-EL. HEK293T cells were transfected with the respective NTSR1 constructs, or with the pcDNA3 plasmid as control. The IP production was recorded using the IP-One HTRF kit. (**a**) Data of constitutive receptor activity assays were normalized for each construct to 100% of the respective value at t = 0 min, and are presented as the inverse of the normalized signal ratio of 665 nm/620 nm. n, number of independent experiments conducted in quadruplicates. NTSR1-WT n = 9 (blue); NTSR1-EL n = 4 (green); NTSR1-ELF n = 6 (magenta); control n = 8 (black). Error bars correspond to s.e.m. (**b**) Agonist-stimulated IP accumulation. Data within each set of dose-response assays were normalized to the largest (100%) and smallest (0%) average value of the respective wild-type dose-response curve, and analysed by nonlinear regression using the GraphPad Prism three-parameter dose-response equation. Data are presented as the inverse of the normalized signal ratio of 665 nm/620 nm. n, number of independent experiments conducted in triplicates: NTSR1-WT n = 5 (blue); NTSR1-EL n = 4 (green); NTSR1-ELF n = 4 (magenta); NTSR1-TM86V n = 4 (grey); control n = 4 (black). Error bars correspond to s.e.m.

NTSR1-EL causes in HEK293T cells inositol phosphate (IP) levels to increase over time in the absence of ligand (Fig. 1). Such a time-dependent increase in IP concentration is greatly reduced with the wild-type receptor^27^ or NTSR1-ELF (Fig. 1). Furthermore, the IP response of NTSR1-EL is almost as large in the absence of ligand compared to saturating concentrations of NTS (Fig. 1). The latter may be underestimated because the constitutive activity of the mutant receptor possibly diminishes the calcium stores of the cells over time. However, NTSR1-EL also activates G protein in response to saturating concentrations of NTS to a much lesser degree than NTSR1-ELF or wild-type NTSR1 in nucleotide exchange experiments using urea-washed insect cell membranes (Supplementary Table 2), an experimental system that assesses the direct interaction of receptor with G protein as opposed to the secondary messenger read-out in intact cells.

For constitutively active mutants, a linear increase of second messenger production is expected with increasing receptor expression levels^13^. As pcDNA3 plasmids carry the SV40 origin, they can replicate in HEK293T cells, which harbour the large T antigen, and thus receptor expression levels are more difficult to control. In addition, different receptor mutants might result in different production levels owing to their specific folding properties and stabilities. We estimated that a receptor density of ∼ 100,000 receptors/cell leads to maximum IP stimulation at saturating concentrations of NTS in HEK293 cells without the large T antigen (Supplementary Fig. 1). Except for NTSR1-Y324A, the NTSR1 mutant expression levels are greater than those of NTSR1-WT in transiently transfected HEK293T cells (Supplementary Table 3). This excess can thus be considered spare receptors not participating in the signalling event.

The ligand binding properties of NTSR1-EL are given in Supplementary Fig. 2 and Supplementary Table 4.

### Architecture of NTSR1-EL

**Figure 2:**
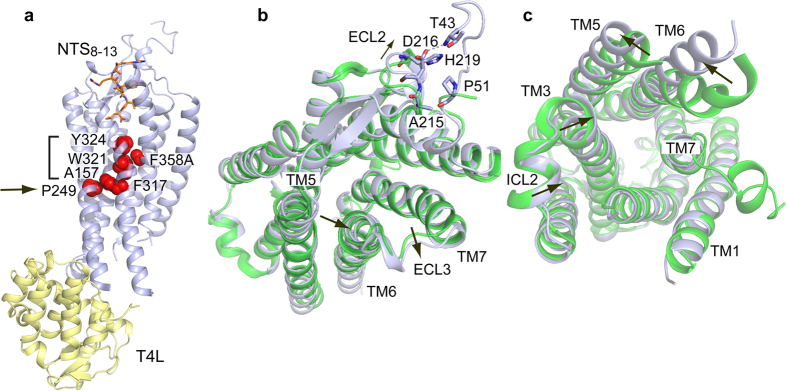
Overview of the NTSR1-EL-T4L structure. (**a**) Side view of NTSR1-EL-T4L in cartoon representation. NTSR1-EL is coloured in grey and T4L in pale yellow. Residues of the hydrophobic cascade (Y324^6.51^, F358^7.42^, W321^6.48^, F317^6.44^, bracket) and the connector region (A157^3.40^, P249^5.50^, F317^6.44^, arrow) are shown as red spheres. NTS_8-13_ is depicted as a stick model (orange). (**b**) Extracellular view. Arrows indicate shifts of TM5, ECL2 and ECL3 in NTSR1-EL-T4L (grey) compared to NTSR1-ELF-T4L (ref. 18) (green). NTS_8-13_ has been omitted for clarity. (**c**) Intracellular view. Arrows indicate the position shift of the intracellular ends of TM3, TM5, TM6 and ICL2 of NTSR1-EL-T4L (grey) compared to NTSR1-ELF-T4L (green).

To understand the structural implications of constitutive activity, we determined the structure of NTSR1-EL to 3.3 Å resolution (Supplementary Table 5, Supplementary Fig. 3). Superposition of NTSR1-EL with NTSR1-ELF reveals that it is similar to this structure (root mean square deviation with a value of 0.6 Å for Cα atoms, excluding T4 lysozyme) indicating an active-intermediate NTSR1 conformation (Fig. 2). The amino (N) terminus of NTSR1-EL extends, compared to NTSR1-ELF, to residue T43^N^ and adopts an unstructured loop that is secured in part by symmetry related molecules. The N-terminus is folded back onto extracellular loop (ECL) 2 promoting van der Waals interactions between P51^N^ and the side chains of A215^ECL2^, D216^ECL2^, and H219^ECL2^. Electrostatic interactions are also seen between the T43^N^ main chain nitrogen and the acidic side chain of D216^ECL2^. Compared to NTSR1-ELF, the loop connecting the ECL2 β-strands has shifted outwards at D216^ECL2^. However, the significance of this observation remains to be established as the position of ECL2 is related to that of the N-terminus, and residues in both the N-terminus and ECL2 are involved in crystal contacts. Compared to NTSR1-ELF, the extracellular end of TM5 of NTSR1-EL is 0.6 Å closer to TM6 when measuring the distances between the Cα positions of T231^5.33^ and C332^6.59^. In addition, the outer tip of TM7 and ECL3 of NTSR1-EL is shifted outwards compared to NTSR1-ELF. On the intracellular side, the ends of most TM segments (TM1-TM3, TM5-TM6) position differently compared to NTSR1-ELF (Fig. 2). In particular, TM5 shows an outward placement away from the receptor core when comparing the V268^5.69^ Cα atom distances of NTSR1-EL and NTSR1-ELF (3.9 Å). TM6 displays an even further outward position when comparing the V300^6.27^ Cα atoms (7.4 Å). The end of TM3 has moved slightly towards the receptor core, which affects the position of ICL2. TM7 of NTSR1-EL terminates at V372^7.56^, thus lacking the unstructured region between TM7 and H8, seen in NTSR1-ELF, and lacking helix 8.

The rotamer position of W321^6.48^, a highly conserved amino acid in class A GPCRs within the CWxP motif, is dictated by the presence or absence of the F358^7.42^ side chain. In NTSR1-ELF with F358^7.42^, the W321^6.48^ side chain is oriented parallel to the lipid bilayer^18^, whereas in NTSR1-EL with the F358A^7.42^ mutation, the W321^6.48^ side chain is oriented perpendicular to the membrane (Supplementary Fig. 4). The same was observed for NTSR1-GW5 (ref. 3) and NTSR1-TM86V-ΔIC3A, which has the F358V^7.42^ mutation^24^.

In NTSR1-ELF, we identified L310^6.37^ as being central to the positioning of the R167^3.50^ side chain such as to allow a *bona fide* productive interaction with the G protein^18^. In NTSR1-EL, both L310^6.37^ and R167^3.50^ side chains are disordered lacking electron density, and so is the E166^3.49^ side chain of the ERY motif.

Subtle, yet distinct differences exist in the NTS_8-13_ binding mode. The movement of TM5 allows the R9 side chain of NTS to engage in a hydrogen bond with T231^5.32^ (Supplementary Table 6) that is absent in NTSR1-ELF. Lacking in NTSR1-EL, but seen in NTSR1-ELF, are hydrogen bond interactions between the NTS carboxylate of L13 and Y351^7.35^ and R328^6.55^. Instead, the NTSR1-EL R328^6.55^ engages in a hydrogen bond contact with N241^5.42^, which is not seen in NTSR1-ELF (Supplementary Fig. 5). Whether the carboxylate of L13 of NTS in NTSR1-EL forms a water-mediated interaction with R328^6.55^ cannot be ascertained at a resolution of 3.3 Å.

### The hydrophobic cascade

**Figure 3:**
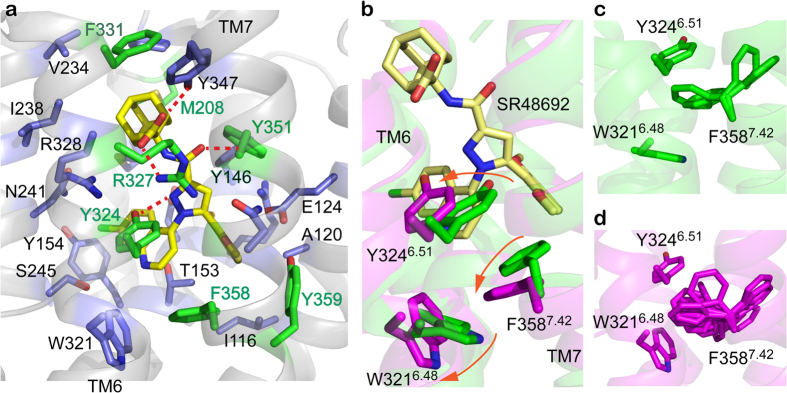
Antagonist docking. (**a**) The predicted binding pocket of the antagonist SR48692 in the NTSR1-SR_dock_ model along with the residues within 4 Å of the ligand are shown. The residues highlighted in green have been experimentally implicated in SR48692 binding^28^. Red dashed lines indicate hydrogen bonds. The predicted binding pose of SR48692 is qualitatively similar to a model proposed previously^28^. However, in our work, the antagonist is located deeper in the binding pocket compared to the model by Labbé-Jullié. The key polar interactions of SR48692 with neurotensin receptor are hydrogen bonds between its carboxylic group and R327^6.54^ and Y347^7.31^. Mutating R327^6.54^ to methionine abolishes SR48692 (and NTS) binding, whereas the Y347A^7.31^ mutation results in loss of agonist affinity without affecting antagonist binding^28^. Y324^6.51^ and Y351^7.35^ form hydrogen bonds with the pyrazole and the amide oxygen of SR48692, respectively. The adamantane ring of SR48692 forms hydrophobic contacts with F331^6.58^ and M208^ECL2^. Both F358^7.42^ and Y359^7.43^ interact with the dimethoxyphenyl moiety of SR48692, whereas Y324^6.51^ engages in π-π stacking with the quinolinyl group of the antagonist. (**b**) Comparison of the side chain orientation of residues Y324^6.51^, W321^6.48^, and F358^7.42^ in the agonist-bound model (NTSR1-NTS_dock_, green) and antagonist-bound model (NTSR1-SR_dock_, magenta). SR48692 is shown as a stick model in yellow. NTS has been omitted for clarity. (**c**,**d**) Side chain rotamer distribution of the residue F358^7.42^ in the agonist and antagonist occupied NTSR1_dock_ models: While F358^7.42^ adopts only a few rotamer conformations in NTSR1-NTS_dock_ (**c**), the rotamer distribution becomes more diverse in NTSR1-SR_dock_ (**d**), indicating the increased mobility of the F358 side chain.

We previously identified in NTSR1-ELF a network of interactions, which we term the hydrophobic cascade, between the NTS binding pocket and the connector. The hydrophobic cascade consists of the residues Y324^6.51^, F358^7.42^, W321^6.48^ and F317^6.44^, whose side chains are in van der Waals contact with each other in NTSR1-ELF (ref. 18). This network is severed in NTSR1-EL owing to the F358A^7.42^ thermostabilizing mutation and concurring perpendicular side chain orientation of W321^6.48^ (Supplementary Fig. 4). As the signalling efficacy of NTS is reduced in NTSR1-EL (Supplementary Table 2), one might speculate that the packing arrangement within the hydrophobic cascade contributes to the communication between the ligand-binding pocket and the connector. In the absence of an inactive, antagonist-bound NTSR1 crystal structure, we performed computer-aided docking of the antagonist SR48692 into the binding pocket of the active-intermediate receptor (NTSR1-SR_dock_) (Fig. 3) to explore the effect on potential structural rearrangements. For comparison, the model NTSR1-NTS_dock_ with NTS_8-13_ bound was generated.

Of significance are the side chain orientations of Y324^6.51^ and F358^7.42^ in the NTSR1-SR_dock_ model (Fig. 3). In the agonist-bound receptor (NTSR1-ELF crystal structure and NTSR1-NTS_dock_), Y324^6.51^, F358^7.42^ and W321^6.48^ form mutual π stacking interactions. In NTSR1-SR_dock_, Y324^6.51^ moves away from TM7 to allow the π-π interaction and hydrogen bond formation with SR48692, thereby disrupting the interaction with F358^7.42^ seen in the agonist-occupied NTSR1. This causes the π stacking between F358^7.42^ and W321^6.48^ to be disrupted resulting in increased mobility of the F358^7.42^ side chain (Fig. 3). W321^6.48^ in turn adopts a side chain orientation perpendicular to the plane of the lipid bilayer. The changed positions of the hydrophobic cascade residues lead to the relocation of the extracellular ends of TM6 and TM7 and a closing of the binding pocket by ECL3. Although the SR48692 docking model presented here is based on an active-intermediate neurotensin receptor crystal structure, it highlights the response of the hydrophobic cascade to different ligands in the binding pocket.

The Y324A^6.51^ mutation yields receptors that show no measurable [^3^H]SR48692 binding^28^ in accordance with the role of Y324^6.51^ in antagonist binding (Fig. 3). The affinity for NTS is not affected in the Y324A^6.51^ mutant^28^. However, the agonist potency appears reduced in IP assays (Supplementary Table 2). Note that the expression level of NTSR1-Y324A is half of that of NTSR1-WT (Supplementary Table 3), and therefore a left-shift of the dose-response curve may occur if the expression level of the mutant would be higher. The W321A^6.48^ mutation in wild type or mutant background also causes only moderate effects on NTS-mediated signalling (Supplementary Table 2). The absence of a strong impact of the Y324A^6.51^ and W321A^6.48^ single mutations on receptor function thus suggests that other transmission links may contribute to the communication between the ligand-binding pocket and the connector in addition to the hydrophobic cascade residues, and that the hydrophobic cascade residues function in parallel, and not in a sequential ‘domino brick’ manner, in the overall allosteric network that effects the conformational changes during the activation process^29^.

### The connector region

The propagation of structural changes in the agonist-occupied binding pocket to the cytoplasmic surface is thought to involve an extensive network of polar interactions^5^ (not discussed here), and conserved residues in the receptor centre, whose packing interactions relate to receptor activation. This region is referred to as the conserved core triad^5^, hydrophobic core^11^ or connector^20^,^30^. Strong packing interactions between I^3.40^, P^5.50^, and F^6.44^ are observed in both the μ-opioid receptor (μOR)^5^ and the β_2_-adrenergic receptor (β_2_AR)^5^,^8^, whereas the packing interactions appear to be reduced in the muscarinic M2 receptor (M2R) with the smaller V111^3.40^ forming weaker contacts to F396^6.44^ and P198^5.50^ (ref. 7). The corresponding residues in NTSR1 are A157^3.40^, P249^5.50^, and F317^6.44^. The conserved residue at position 3.40 is large and hydrophobic in over 70% of all class A GPCRs^11^. However, in NTSR1 it is an alanine (A157^3.40^) with a much smaller side chain than that of an isoleucine in the β_2_AR and μOR. Consequently, one would anticipate more subtle connector packing differences between NTSR1 conformational states compared to β_2_AR or μOR. The connector regions of the NTSR1-EL and NTSR1-ELF crystal structures are, as expected, similar, despite the efficacy differences of NTS for both constructs (Fig. 1). However, we note in NTSR1-EL weak van der Waals contacts between the CE1 and CZ atoms of F317^6.44^ and the CA and CB atoms of A157^3.40^, whereas no such interactions are found between F317^6.44^ and A157^3.40^ in the active-intermediate NTSR1-ELF structure. In addition, TM3 of NTSR1-EL is slightly shifted towards the receptor centre (Supplementary Fig. 6).

The rotamer orientation of the W321^6.48^ side chain in the active-intermediate NTSR1 crystal structures impacts upon the distance between TM3 and TM6 within the connector (Supplementary Table 7). The perpendicular orientation of W321^6.48^ allows tighter packing (reduced distance, NTSR1-EL and NTSR1-GW5), whereas the parallel orientation keeps TM3 and TM6 further apart (NTSR1-ELF and NTSR1-LF). However, this effect is less pronounced for NTSR1_MD_ (Supplementary Table 8).

In the crystal structures of active-intermediate NTSR1 (refs. 3 and 18) and TM86V-ΔIC3A^24^, the packing interactions between the P249^5.50^-A157^3.40^ and A157^3.40^-F317^6.44^ side chains show comparatively small changes. This is in contrast to the distance between the F317^6.44^ and P249^5.50^ side chains, with TM86V-ΔIC3A displaying the largest distance among the various NTSR1 constructs (Supplementary Table 9). TM86V-ΔIC3A is a NTSR1 mutant that adopts an apparent inactive receptor conformation at the inner side in the crystal structure^24^, but it is uncertain whether its connector region represents a fully inactive state. In the NTSR1 crystal structures, the inter-residue side chain distance between P249^5.50^ and F317^6.44^ is smaller for all active-intermediate neurotensin receptors compared to TM86V-ΔIC3A (Supplementary Table 9). Comparison of the distances between F^6.44^ and P^5.50^ in the crystal structures of inactive and active β_2_AR, M2R and μOR also indicates closer packing between those residues in the active state (Supplementary Table 9).

**Figure 4:**
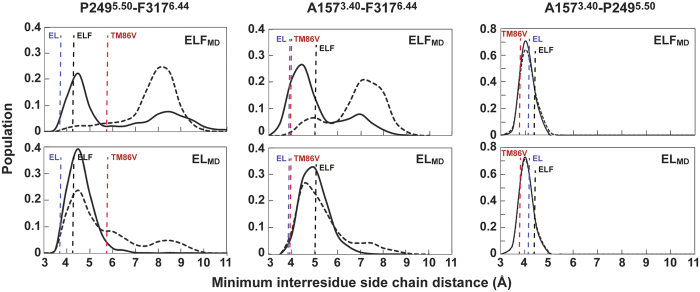
Packing of connector residues during MD simulations. Minimum inter-residue distances between any pair of the side chain atoms (excluding hydrogen atoms) of A157^3.40^, P249^5.50^ and F317^6.44^ were measured over the entire MD trajectories for NTSR1-ELF_MD_ (top) and NTSR1-EL_MD_ (bottom) ensembles in the presence of NTS (solid curves) and absence of ligand (dashed curves). The vertical lines represent the minimum inter-residue side chain distances in the NTSR1-EL-T4L (PDB code 5T04, blue), NTSR1-ELF-T4L (PDB code 4XEE, black) and TM86V-ΔIC3A (PDB code 3ZEV, red) crystal structures.

Crystallization attempts of NTSR1-EL in the absence of NTS_8-13_ were unsuccessful. Therefore, we used MD simulations (the energetics of NTSR1_MD_ are shown in Supplementary Fig. 7 and Supplementary Table 10) to analyse the variation of the minimum inter-residue distances between connector residues of NTSR1-EL_MD_ and NTSR1-ELF_MD_ in the presence and absence of agonist (Fig. 4).

In the presence of NTS, the packing interactions between P249^5.50^, A157^3.40^ and F317^6.44^ in NTSR1-EL_MD_ and NTSR1-ELF_MD_ are similar to that seen in the crystal structures of the agonist-occupied NTSR1-EL and NTSR1-ELF. This does not substantially change for NTSR1-EL_MD_ in the absence of NTS. However, the NTSR1-ELF_MD_ system shifts to a much larger distance in the absence of agonist (the peaks of the distribution for the P249^5.50^-F317^6.44^ and A157^3.40^-F317^6.44^ distances centre at 7–8 Å rather than 4–5 Å). Thus the connector region of NTSR1-EL_MD_ appears ‘active’ irrespective of the presence or absence of agonist. This is in contrast to NTSR1-ELF_MD_, where a more compact connector region is only observed in the presence of NTS in the ligand-binding pocket. Assuming that closer packing of connector residues is indicative for a more active state (Fig. 4), the dynamic behaviour of NTSR1-ELF_MD_ might explain why nucleotide exchange at the G protein occurs in response to agonist, but no appreciable constitutive activity is seen in the absence of NTS. In contrast, the connector region of NTSR1-EL_MD_ remains packed in the presence and absence of NTS, possibly explaining its constitutive activity and the reduced effect of NTS on signalling.

### Dynamics of W321^6.48^ and F317^6.44^

Spectroscopic evidence suggested changes in the environment of W^6.48^ upon rhodopsin activation^31^,^32^. However, a rotamer change of W^6.48^ was not observed in any crystal structure of active GPCRs^5^,^7^,^8^,^9^,^33^, but only found in the structures of active-intermediate NTSR1-ELF and NTSR1-LF^18^. MD simulations based on the active μOR structure^5^ suggested that W^6.48^ serves as a link between the ligand binding pocket and the connector region.

**Figure 5:**
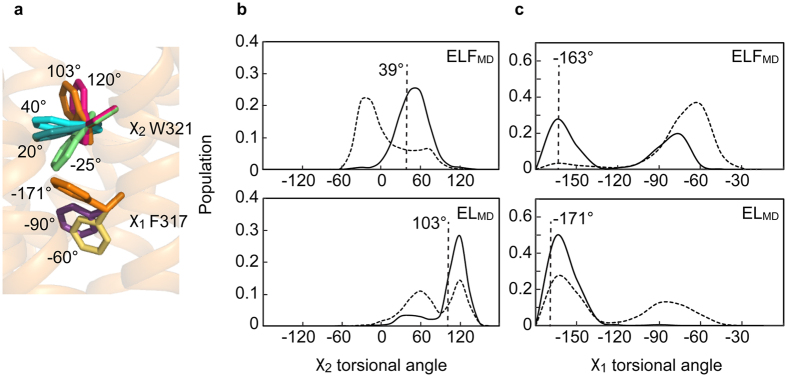
Dynamic behavior of the W321^6.48^ and F317^6.44^ residues. (**a**) Hypothetical side chain orientations of W321^6.48^ (χ_2_ angles of 120°, 40°, 20°, −25°) and F317^6.44^ (χ_1_ angles of −90°, −60°) are projected onto the NTSR1-EL-T4L crystal structure (orange, W321^6.48^ χ_2_ angle of 103°, F317^6.44^ χ_1_ angle of −171°). (**b**) The population distribution of the χ_2_ torsional angles of W321^6.48^ for NTSR1-ELF_MD_ (top panel) and NTSR1-EL_MD_ (bottom panel) over the entire MD trajectory. The vertical lines represent the χ_2_ angles in the respective crystal structures. (**c**) The population distribution of the χ_1_ angles of F317^6.44^ for NTSR1-ELF_MD_ and NTSR1-EL_MD_. The vertical lines represent the χ_1_ angles in the respective crystal structures. Solid curves are population distributions in the presence of agonist, whereas hashed curves are population distributions in the absence of agonist.

Crystallography typically captures the lowest energy state within an ensemble of conformations (here active-intermediate) that may subtly differ from other signalling conformations of membrane-inserted receptors, not observed in crystal structures^21^. Therefore, we studied the dynamic behaviour of W321^6.48^ and F317^6.44^ by MD simulations. The χ_2_ angles of W321^6.48^ of NTS-occupied NTSR1-ELF_MD_ and NTSR1-EL_MD_ stay close to the respective angles in the corresponding crystal structures (Fig. 5). In contrast, the W321^6.48^ side chain becomes more dynamic in the absence of agonist, adopting perpendicular and parallel rotamer orientations in NTSR1-EL_MD_, and tilting even further towards the intracellular receptor side in NTSR1-ELF_MD_ (χ_2_ angle of −25°). This is accompanied by a change of the F317^6.44^ side chain orientation to avoid steric hindrance, as the benzene moiety of the W321^6.48^ indol ring would come too close to the phenyl side chain of F317^6.44^ as seen in the NTSR1-ELF and NTSR1-EL crystal structures (χ_1_ torsional angles of −163° and −171°, respectively). The change of the F317^6.44^ side chain position (χ_1_ angle change from −163° to −60°) would resolve the clash (Fig. 5).

In addition, the mobility of the F317^6.44^ side chain appears also to be an inherent property, and not solely determined by the rotamer orientation of W321^6.48^, as F317^6.44^ adopts side chain positions with χ_1_ angles ranging from −90° to −60°, when the W321^6.48^ side chain is oriented parallel (agonist-occupied NTSR1-ELF_MD_, χ_2_ angle of 55°) and perpendicular (NTSR1-EL_MD_ without NTS, χ_2_ angle of 120°).

### Conformational dynamics of the G protein coupling interface

Activation of the G protein by an agonist-occupied receptor or by a constitutively active receptor mutant requires an initial docking step of the G protein, which may be facilitated by a more open cleft and higher flexibility within the intracellular face of the receptor.

The structure of agonist-bound NTSR1-EL appears more open at the intracellular side than that of NTSR1-ELF (Fig. 2), suggesting easier accommodation of the C-terminus of a Gα subunit, yet the efficacy of NTS in IP assays is less than that for NTSR1-ELF (Fig. 1). However, the distance between residues N257^5.58^ and Y369^7.53^, whose interaction was implied to stabilize the active receptor conformation^7^, is larger in the NTSR1-EL crystal structure compared to NTSR1-ELF (Supplementary Table 11), suggesting possibly a less active-intermediate state for NTSR1-EL, in agreement with the pharmacology of the respective mutants. As crystal structures of NTSR1 in complex with G protein are currently unavailable for comparison, we used MD simulations to assess the receptor dynamics at the G protein interface.

**Figure 6:**
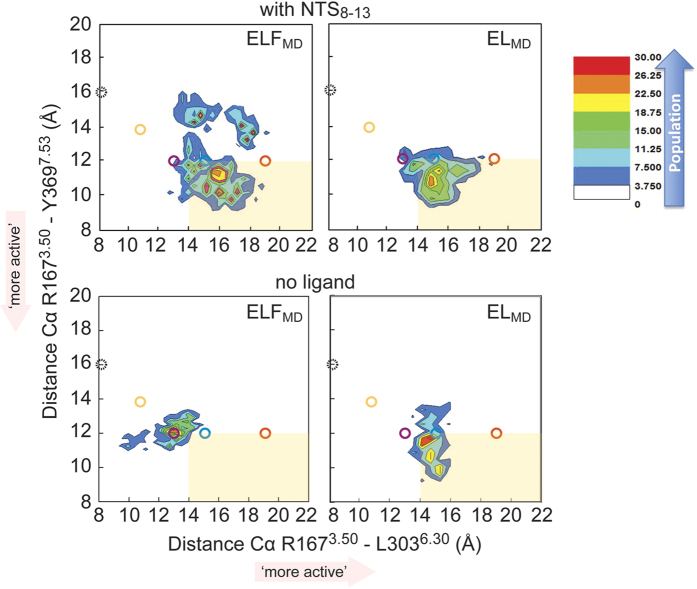
Conformational dynamics of the G protein-coupling interface. NTSR1_MD_ snapshots were sampled in MD simulations and projected into the distance between TM3 and TM6, and the distance between TM3 and TM7. Contour maps of the Cα-Cα distances of R167^3.50^-L303^6.30^ and R167^3.50^-Y369^7.53^ are shown for NTSR1-ELF_MD_ and NTSR1-EL_MD_ in the presence of NTS_8-13_ (upper panels) and absence of ligand (lower panels). The conformational clusters are colour coded from blue to red according to their population density for the TM3-TM6 and TM3-TM7 distances, with red indicating larger populations in the density distributions. Open circles represent the Cα-Cα distances found in the crystal structures of inactive NTSR1 (PDB code 3ZEV, black); inactive β_2_AR (PDB code 2RH1, orange); active β_2_AR (PDB code 3SN6, red); NTSR1-ELF-T4L (PDB code 4XEE, magenta) and NTSR1-EL-T4L (PDB code 5T04, blue). The light orange box indicates ‘more active’ distances between TM3-TM6 and TM3 and TM7.

We compared ensembles of conformations of NTSR1_MD_, with and without NTS, using distance measurements between TM3-TM6 (distance between Cα atoms of R167^3.50^-L303^6.30^) and TM3-TM7 (distance between Cα atoms of R167^3.50^-Y369^7.53^) (Fig. 6). An increase in the distance between TM3 and TM6, and a decrease in the distance between TM3 and TM7, is observed in the active state structures of other class A GPCRs (for example ref. 9). Thus the distance measurements between TM3-TM6 and TM3-TM7 may serve as one of the indicators for the activation state of the receptor. The ensemble clusters show two distinct populations (which we call ‘less’ active-intermediate and ‘more’ active-intermediate) for NTSR1-ELF_MD_ in the presence of agonist, whereas NTSR1-EL_MD_ displays a more homogenous single ensemble in the presence of the peptide (Fig. 6). Analysis of the population density distribution of the TM3-TM6 and TM3-TM7 distances in the absence of agonist shows reduced heterogeneity for NTSR1-ELF_MD_. For NTSR1-EL_MD_, the differences between NTS-loaded and agonist-free population distributions are small with the ensembles (both with and without ligand) being ‘more’ active-intermediate than those of NTSR1-ELF_MD_ in the absence of agonist. The appearance of distinct populations for NTSR1-ELF_MD_ in the presence of NTS correlates with multiphasic dose response curves in nucleotide exchange assays for NTSR1-ELF, whereas the more homogenous single ensemble of NTSR1-EL_MD_ relates to the monophasic behaviour for NTSR1-EL (Supplementary Table 2).

**Figure 7:**
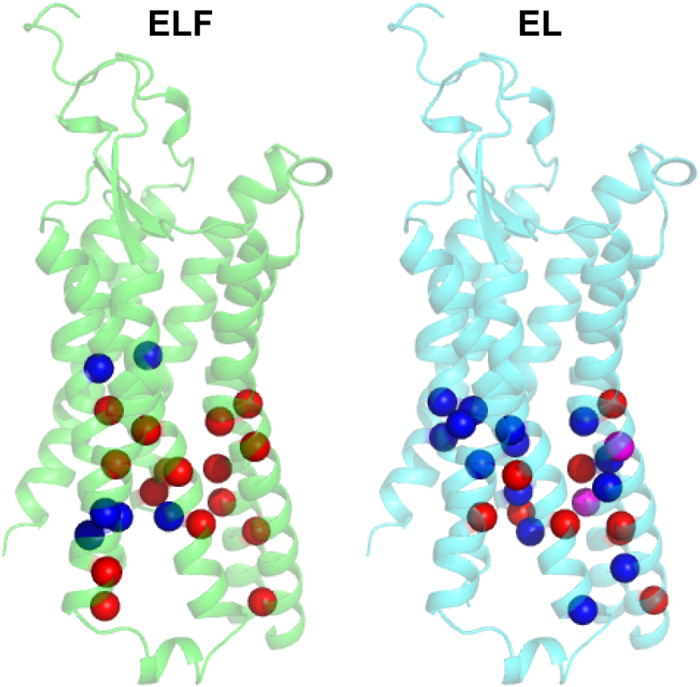
Interhelical contacts of NTSR1_MD_. Interhelical hydrogen bond and van der Waals interactions that are present in more than 50% of the conformations in the MD trajectories were identified (Supplementary Table 12). Spheres represent the Cα atoms of residues engaged in interhelical contacts, projected onto the NTSR1-EL-T4L crystal structure. Interactions in the presence and absence of NTS are coded in blue and red, respectively, and are shown for NTSR1-ELF_MD_ (left) and NTSR1-EL_MD_ (right). Residues marked in magenta are found in both MD simulations (presence and absence of agonist) of NTSR1-EL_MD_, but those residues engage in different contacts. Interhelical contacts that are identical in the MD simulations in the presence and absence of agonist for NTSR1-ELF_MD_ and NTSR1-EL_MD_, respectively, are not shown.

The strength of interhelical interactions relates to the flexibility within the receptor, possibly influencing the initial encounter between receptor and G protein. We therefore calculated the hydrogen bond and van der Waals contacts in NTSR1_MD_ (Supplementary Table 12). NTSR1-ELF_MD_ shows stronger interhelical packing i.e. more interhelical contacts in the absence of NTS within the intracellular receptor half compared to the presence of agonist. The opposite is observed for NTSR1-EL_MD_ where there are fewer interactions i.e. weaker interhelical packing in the absence of NTS compared to the presence of agonist. Notably, the location of many interhelical contacts within the respective conformational ensembles (NTSR1-ELF_MD_ or NTSR1-EL_MD_, with and without agonist) is different (Fig. 7), although a number of interactions are identical.

The stimulation by NTS of a signalling response is high for NTSR1-ELF, but modest for NTSR1-EL (‘fold stimulation’, see Supplementary Table 2). In contrast, NTSR1-EL signals in the absence of agonist, which NTSR1-ELF almost does not (Fig. 1). Overall, the analysis of the conformational dynamics of the G protein interface of NTSR1_MD_ suggests higher flexibility (Fig. 7, Supplementary Table 12) and the propensity to adopt ‘more active’ active-intermediate states (Fig. 6) as possible parameters for the ability to accommodate the incoming G protein.

## Discussion

Constitutively active receptor mutants are thought to adopt spontaneously a conformation able to activate G protein, possibly by either releasing inactive state constraints and/or forming new interactions that stabilize a more active state^13^, lowering the energy barrier between the inactive and active receptor conformations. Constitutively active mutants have been described for many GPCRs^34^. However, the structural basis of constitutive activity has only been investigated for a limited number of rhodopsin mutants targeting the retinal binding pocket^33^,^35^ and the G protein-binding site^36^. In the E113Q^3.28^ rhodopsin mutant structure^33^, the salt bridge between E113^3.28^, as counter-ion to the protonated Schiff base, and K296^7.43^ is broken, removing constraints between TM3 and TM7 in the inactive state. The G90D^2.57^ mutation introduces a stabilizing salt bridge to K296^7.43^, which disfavours covalent binding of the retinal ligand and destabilizes the inactive conformation^35^. The M257Y^6.40^ mutation stabilizes the G protein-binding interface through interactions with residues that are critical for receptor activation (Y306^7.53^, Y223^5.58^, R135^3.50^) (ref. 36). Furthermore, the crystal structure of the human cytomegalovirus GPCR US28 in complex with fractalkine in an active-intermediate state has been reported^37^ and MD simulations suggested that the agonist-independent activity of US28 may result from amino acid interactions of the DRY motif destabilizing the inactive state. The rhodopsin mutants provide examples in which the molecular basis for constitutive activity is rather specific^36^. The explanation of inherent constitutive activity of many other receptors, likely originating from an overall increased conformational flexibility, may thus become more complex than that for the rhodopsin mutants.

The NTSR1 sequence contains a bulky phenylalanine residue at position 7.42 and a tryptophan residue at position 6.48. The combination of F^7.42^ and W^6.48^ is also found in the receptors for neuromedin U, motilin and ghrelin, and NTSR2, but not in other class A GPCRs^18^. The motilin receptor and wild-type NTSR1 are silent with respect to constitutive activity^27^,^38^. In contrast, the ghrelin receptor is highly constitutively active and has been extensively characterized by pharmacological, mutagenesis and modelling studies^29^,^38^,^39^. Constitutive activity has also been ascribed to the single point mutant NTSR1-F358A^27^, but no crystal structure has been reported for this mutant.

Why is NTSR1-EL constitutively active? The molecular basis for constitutive activity and ligand-based activation of NTSR1 is expected to be similar as it ultimately results in the binding and activation of G protein in both cases. Therefore, the comparison of NTSR1-EL, which signals both in the presence and absence of NTS, with NTSR1-ELF, which causes a secondary messenger response only in the presence of agonist (Fig. 1), may point to features in the connector region and the intracellular receptor face associated with activation. The connector regions of the NTSR1-EL and NTSR1-ELF crystal structures do not show large differences in the overall packing arrangement, which is perhaps anticipated, as both structures represent active-intermediate conformations. However, we observe in MD simulations tight contacts of connector residue side chains in NTSR1-EL_MD_, irrespective of the presence or absence of agonist, as is also found in NTS-occupied NTSR1-ELF_MD_, but not in ligand-free NTSR1-ELF_MD_ (Fig. 4). Thus tight interactions within the connector region may describe a conformational element attributed to the activation of NTSR1, which correlates with significant IP production of NTSR1-EL without and with agonist, and NTSR1-ELF with NTS (Fig. 1). A more open cleft (Fig. 6) and fewer interhelical contacts within the intracellular half of the receptor (Fig. 7, Supplementary Table 12) may accelerate the initial contact between G protein and NTSR1-ELF in the presence of agonist, and NTSR1-EL irrespective of ligand occupancy.

What are the global changes within the connector region upon activation? Although the crystal structure of NTSR1 in its inactive, antagonist-bound state is currently unknown, comparison of the agonist-bound NTSR1-EL and TM86V-ΔIC3A structures reveals distinct differences within the connector region (Supplementary Fig. 8). TM86V-ΔIC3A is a NTSR1 mutant that has been crystallized in the presence of NTS but adopts an apparent inactive receptor conformation at the inner receptor side^24^ and does not produce an IP signalling response (Fig. 1). The W321^6.48^ side chain in TM86V-ΔIC3A is positioned upward, like in NTSR1-EL, because of the F358V^7.42^ mutation. In NTSR1-EL, TM3 is shifted up compared to TM86V-ΔIC3A, and TM3 bulges inwards at T156^3.39^, V160^3.43^, and S164^3.47^. TM5 bulges slightly in towards TM3 at P249^5.50^ and S245^5.46^ (one turn towards the ligand binding pocket) in the NTSR1-EL structure. The counter-clockwise rotation of F317^6.44^ (viewed from the top) causes the distance between the P249^5.50^–F317^6.44^ side chains to become smaller in NTSR1-EL (Supplementary Table 9).

**Figure 8:**
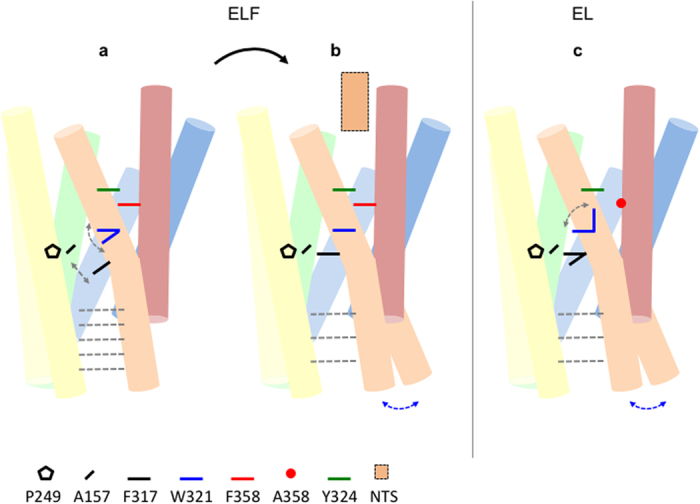
Constitutive and agonist-induced signalling of NTSR1. The cartoons compare ‘activation’ features of (**a**) NTSR1-ELF in the absence of agonist, (**b**) NTSR1-ELF with NTS, and (**c**) NTSR1-EL without ligand. The schematic, oversimplified representation is based on crystallographic data and MD simulations. Amino acids of the hydrophobic cascade and the connector region are shown as solid coloured symbols, and NTS is depicted as an orange box. Dashed grey lines indicate the relative number of interhelical contacts within the intracellular half of the receptor, and dashed grey arrows indicate side chain mobility. Blue arrows indicate a higher propensity of the receptor to accommodate the incoming G protein. The overall structure of the unliganded receptor is assumed to be similar, but not identical, to that of the NTS_8-13_ bound NTRSR1-EL structure because the IP response is almost as large in the absence of ligand compared to saturating concentrations of NTS, and the dynamic properties of NTSR1-EL_MD_ are similar with and without agonist. A network of amino acid side chain interactions constitutes the link between the NTS-occupied binding pocket and the connector region. This interaction is disrupted in NTSR1-EL, possibly explaining the diminished signalling potency of NTS. The rotamer orientation and dynamics of the conserved W321^6.48^ side chain affect neighbouring amino acid residues, and are relevant to the activation process as this determines the position of transmembrane helices and the packing of the connector, implicated in receptor activation. Tightly packed residues of the connector and a flexible G protein interface may represent switches in intermediate conformations, facilitating the initial interaction with the G protein and explaining the constitutive activity of NTSR1-EL.

What is the role of W321^6.48^ and the hydrophobic cascade in the signalling process of NTSR1? Although W^6.48^ is highly conserved in class A GPCRs, about one fifth of receptors have another aromatic or non-aromatic residue at this position^39^. Furthermore, mutation of W^6.48^ to alanine and other residues resulted in strong effects on signalling in some receptors, but showed no or little consequence in others. For example, mutation to alanine of W^6.48^ in the ghrelin receptor eliminated constitutive activity and strongly impaired agonist-induced signalling^39^, whereas alanine substitution in NTSR1 has only moderate effects (Supplementary Table 2). These observations suggest that the role of W^6.48^ in the signal transduction mechanism is receptor dependent. In NTSR1, the rotamer orientation of the W321^6.48^ side chain and the integrity or disconnect of the hydrophobic cascade appear to relate to agonist sensing. NTS-occupied NTSR1-ELF, which has stacked hydrophobic cascade residues and the W321^6.48^ side chain in a parallel orientation, signals almost as strong as the wild-type receptor. In contrast, NTSR1-EL, with a perpendicular W321^6.48^ side chain orientation and a small non-aromatic alanine at position 7.42, shows a strongly reduced response above basal in the presence of agonist. The inability of NTS to stimulate a signalling response in NTSR1-LF, which has the W321^6.48^ side chain in a parallel orientation in the crystal structure^18^, may have its cause in the E166A^3.49^ mutation in the conserved ERY motif. There are no crystal structures of any un-liganded NTSR1 mutants to examine the W321^6.48^ side chain orientations, but increased mobility of W321^6.48^ is observed in MD simulations in the absence of agonist. In addition, substantial rearrangements and increased dynamics of hydrophobic cascade residues are seen in the docking model NTSR1-SR_dock_ with antagonist bound (Fig. 3). Thus it appears that NTSR1 communicates the presence of NTS to the connector region by stacking of the hydrophobic cascade residues. Severing this link in NTSR1-EL reduces the sensing of bound agonist and thus peptide-induced signalling (Fig. 8).

## Methods

### NTSR1 constructs

The baculovirus construct NTSR1-EL-T4L used for crystallization consisted of the hemagglutinin signal peptide and the Flag tag^40^, followed by the thermostabilized rat NTSR1 (T43-K396 containing the mutations A86L^1.54^, G215A^ECL2^, F358A^7.42^, V360A^7.44^) with the ICL3 residues H269-E296 replaced by the cysteine-free bacteriophage T4 lysozyme (N2-Y161 with the mutations C54T and C97A) and a GSGS linker. A deca-histidine tag was placed at the C-terminus. All other constructs used for pharmacological analyses are summarized in Supplementary Table 1.

In the Methods sections, we distinguish between NTSR1 constructs containing T4L or the wild-type ICL3 sequence. In the main text, we use only one name for a particular construct; NTSR1-EL refers interchangeably to NTSR1-EL-T4L and NTSR1-EL, the latter containing the wild-type ICL3, not T4L. The identity of the respective construct is evident from the context of writing.

**Expression of Gq protein in insect cells and purification** was done as described^18^.

**Expression of NTSR1 in insect cells** was done as described^18^.

**Preparation of urea-washed P2 insect cell membranes** was done as described^18^.

### Ligand binding experiments

All radioligand binding assays were conducted as described^18^ with urea-washed P2 insect cell membranes containing the indicated NTSR1 constructs. Independent experiments were carried out in single data points.

Data of agonist [^3^H]NTS ([3,11-tyrosyl-3,5-3 H(N)]-pyroGlu-Leu-Tyr-Glu-Asn-Lys-Pro-Arg-Arg-Pro-Tyr-Ile-Leu) (PerkinElmer) saturation binding experiments were analysed by nonlinear regression using the GraphPad Prism software and best fit to a one-site binding equation to determine the dissociation constants (K_d_). Note that the saturation binding experiments using wild-type NTSR1 were conducted at equilibrium. In contrast, binding of [^3^H]NTS to the NTSR1 mutants did not reach equilibrium within the incubation time because of the slow agonist off-rates.

Competition assays with the non-peptide antagonist SR48692 (ref. 26) were performed in the presence of [^3^H]NTS. Data were analysed by nonlinear regression with the GraphPad Prism three-parameter dose-response equation using the concentrations of total SR48692 added versus bound [^3^H]NTS. IC_50_ values were converted into K_i_ values using the Cheng Prusoff equation^41^.

The effect of Na^+^ ions on [^3^H]NTS binding was recorded and data were analysed by nonlinear regression using the GraphPad Prism four-parameter dose-response equation (variable slope) with the top and bottom plateau constrained from 100–50% (NTSR1-EL-T4L, NTSR1-EL).

The association and dissociation of [^3^H]NTS was assessed as described^18^.

### GTPγS assays

Prior to G protein coupling assays, the P2 membranes were treated with urea to remove peripherally bound membrane proteins^42^,^43^. GDP/[^35^S]GTPγS exchange assays were performed as described^18^,^44^,^45^ with 1 nM receptor, 140 nM Gq protein, and specified amounts of NTS in the reaction. Experiments were conducted either at saturating ligand concentrations (NTS at 20 μM) or using a range of ligand concentrations for dose-response assays (0–20 μM). Independent experiments were carried out in single data points. Data from dose-response experiments were fit to an equation with a Hill slope of 1, except when indicated otherwise.

### Inositol phosphate determination

HEK293T or HEK293 cells were transiently transfected with pcDNA3 or pME-HA (Lucigen) plasmid derivatives (Supplementary Table 1) using the lipofectamine 3000 reagent (Invitrogen). As a control, cells were transfected with the parental pcDNA3 vector.

For inositol phosphate determination, HEK293T or HEK293 cells were seeded the day after transfection in white-walled 96 well plates (Thermo Scientific) (100 μl per well; 50,000 cells/well). The next day, IP accumulation was measured using the IP-One HTRF kit (Cisbio Bioassays) according to the manufacturer’s instructions. The IP One assay is a competitive immunoassay based on an IP_1_-specific antibody labelled with Lumi4-Tb cryptate. IP_1_ produced by cells competes with d2-labeled IP_1_ for binding to the antibody. Thus the signal is inversely proportional to the level of cellular IP. Cells were stimulated for 60 min at 37 °C with NTS (100 pM – 10 μM) in 70 μl of IP stimulation buffer containing LiCl to inhibit the degradation of IP. To probe for constitutive receptor activity, cells were incubated for the indicated time points in the absence of ligand. IP_1_-d2 reagent (15 μl) and an anti-IP_1_ antibody labelled with Lumi4-Tb cryptate (15 μl) were then added to the cells. The mixture was incubated for 1 hour at room temperature. Signals at 665 nm and 620 nm were detected using a FlexStation 3 (Molecular Devices) fluorescence reader, and values were expressed as the signal ratio of 665 nm / 620 nm. Independent experiments were carried out in triplicates (does-response experiments) or quadruplicates (constitutive receptor activity). Data within each set of dose-response assays were normalized to the largest (100%) and smallest (0%) average value of the respective wild-type dose-response curve, and analysed by nonlinear regression using the GraphPad Prism three-parameter dose-response equation. Data are presented in figures as the inverse of the normalized signal ratio of 665 nm/620 nm. Data of constitutive receptor activity assays were normalized for each construct to 100% of the respective value at t = 0 min. Data are presented in figures as the inverse of the normalized signal ratio of 665 nm/620 nm.

To determine the minimum number of receptors saturating the IP response at 10 μM NTS, HEK293 cells were transfected with varying amounts of plasmid. The day after, the cells were seeded in white-walled 96 well plates (Thermo Scientific) (100 μl per well; 50,000 cells/well, quadruplicates) and in 6 well plates (2 ml per well; 1 × 10^6^ cells/well, duplicates). The next day, cells in 96 well plates were probed for IP production for 60 min as described above. Data in the presence of NTS were normalized for each plasmid concentration to 100% of the respective value at t = 0 min. Data are presented in figures as the inverse of the normalized signal ratio of 665 nm/620 nm. The cells in the 6 well plates were detached, suspended in PBS, and subjected to radioligand binding analysis at 10 nM [^3^H]NTS. The cell density was determined using the Countess II FL automated cell counter (Invitrogen), and the receptor number at the cell surface was calculated. The amount of specifically bound [^3^H]NTS (in dpm) in 96 well plates was estimated from the amount of specifically bound [^3^H]NTS in 6 well plates by dividing this number by 20 (1 × 10^6^/50,000).

### Purification of NTSR1-EL-T4L

Cells from 3 L of insect cell culture were thawed and the volume was brought to approximately 350 ml with hypotonic buffer (10 mM Hepes pH 7.5, 10 mM MgCl_2_, 20 mM KCl). The cells were then resuspended using a Turrax T-25 (IKA) homogenizer at 8,000 rpm for 2 min. After centrifugation (45Ti rotor, 125,000× g, 20 min, 4 °C, Optima L90K, Beckman), the membranes were resuspended (Turrax T-25) in approx. 350 ml of high-salt buffer (10 mM Hepes pH 7.5, 1 M NaCl, 10 mM MgCl_2_, 20 mM KCl) supplemented with AEBSF (100 μM), and centrifuged again. The high-salt buffer wash was repeated one more time resulting in about 22 gram of wet membrane pellet. All subsequent steps were performed at 4 °C or on ice, and AEBSF (100 μM final concentration) was repeatedly added throughout the procedure. The washed membranes were resuspended in 187 ml of buffer [75 mM TrisHCl pH 7.4, 45% (v/v) glycerol] containing 15 μM NTS_8-13_ (Arg-Arg-Pro-Tyr-Ile-Leu) and stirred for 30 min to allow agonist binding to membrane-inserted NTSR1. The receptor was extracted by drop-wise addition of 93 ml of a 3% (w/v) lauryl maltose neopentyl glycol (2,2-didecylpropane-1,3-bis-β-D-maltopyranoside) (LMNG) (Anatrace)/0.3% (w/v) cholesteryl hemisuccinate Tris salt (CHS) (Anatrace) solution. After 2 hours, NaCl was added, and the solution was gently stirred for an additional 15 min. The final volume was 280 ml containing 50 mM TrisHCl pH 7.4, 30% (v/v) glycerol, 200 mM NaCl, 1% (w/v) LMNG/0.1% (w/v) CHS and 10 μM NTS_8-13_. The sample was clarified by centrifugation (45Ti rotor, 125,000× g, 1 hour, Optima L90K, Beckman), adjusted with imidazole to a final concentration of 20 mM, and batch-incubated overnight with 1.5 ml Talon resin equilibrated with Talon-A^+^ buffer [50 mM TrisHCl pH 7.4, 30% (v/v) glycerol, 200 mM NaCl, 20 mM imidazole, 1 μM NTS_8-13_, 0.1% (w/v) LMNG/0.01% (w/v) CHS]. After washing the resin with 22.5 ml of buffer Talon-A^+^ and 15 ml of buffer Talon-A2^+^ [50 mM TrisHCl pH 7.4, 30% (v/v) glycerol, 200 mM NaCl, 20 mM imidazole, 1 μM NTS_8-13_, 0.05% (w/v) LMNG/0.005% (w/v) CHS], NTSR1-EL-T4L was eluted in 0.5 ml steps with Talon-B^+^ buffer [50 mM TrisHCl pH 7.4, 30% (v/v) glycerol, 200 mM NaCl, 250 mM imidazole, 10 μM NTS_8-13_, 0.05% (w/v) LMNG/0.005% (w/v) CHS]. Peak fractions were collected (2.5 ml) and desalted using a PD10 column equilibrated in PD10 buffer [50 mM TrisHCl pH 7.4, 200 mM NaCl, 0.003% (w/v) LMNG/0.0003% (w/v) CHS]. NTS_8-13_ was then added to a concentration of 20 μM, and the sample was used for crystallization. Three liters of insect cell culture yielded ~3.3 mg of purified NTSR1-EL-T4L.

### Stability tests in detergent solution

The stability of NTSR1 was measured in the presence of LMNG/CHS in the +NTS format^45^ as described^18^. Control reactions on ice were recorded at the start and at the end of each denaturation experiment. The percentage of activity remaining after heat exposure was determined with respect to the unheated control. Data were analysed by nonlinear regression using a Boltzmann sigmoidal equation in the Prism software (GraphPad).

### Crystallization

Purified desalted NTSR1-EL-T4L was adjusted to 100 μM Tris (2-carboxyethyl) phosphine hydrochloride (TCEP) and 350 μM NTS_8-13_ and concentrated to an estimated 60 mg ml^−1^ using a 100,000 MWCO concentrator (Amicon Ultra, Millipore). After addition of NTS_8-13_ to 1.5 mM and centrifugation (TLA 120.1 rotor, 128,000× g, 30 min, 4 °C, Beckman), the sample was mixed with 1.5 parts by weight of a mix of monoolein with cholesterol (10:1) using the two-syringe method^46^. The resulting lipidic cubic phase^47^ mix was dispensed in 60–70 nl drops onto Laminex plates (Molecular Dimensions) and overlaid with 825 nl precipitant solution using a Mosquito LCP robot (TTP Labtech). Crystals of NTSR1-EL-T4L grew at 20 °C after 3 days in precipitant solution consisting of 13–16% (v/v) PEG 400, 80 mM TrisHCl pH 8.5–9.0, 1.9 mM TCEP, 68–91 mM lithium acetate and 0.9 mM NTS_8-13_. Crystals were harvested directly from LCP using micro-loops (MiTeGen) and immediately flash frozen in liquid nitrogen without adding extra cryoprotectant.

### Data collection and structure determination

Data collection was performed using the JBluIce-EPICS data acquisition software at the GM/CA-CAT (23-ID-B and 23-ID-D) beamlines at the Advanced Photon Source of the Argonne National Laboratory using a 10–20 μm minibeam at a wavelength of 1.0332 Å. Crystals within the loops were located by diffraction using the automated rastering module of JBluIce-EPICS^48^,^49^. Partial data sets (wedges of 5–10 degrees) were collected from crystals exposed to the non-attenuated minibeam for 1–2 sec and 1 degree oscillation (23-ID-B), or 0.3 sec and 0.3 degree oscillation (23-ID-D) per exposure.

For NTSR1-EL-T4L, a 97.3% complete data set at 3.3 Å resolution was obtained by indexing, integrating, scaling, and merging partial data sets from 13 crystals using XDS^50^, and Aimless^51^ of the CCP4 Suite^52^.

Structure determination was performed by molecular replacement using the Phaser module of the CCP4 Suite. Two search models were created using the structure of NTSR1-ELF-T4L (PDB code 4XEE) with one containing the T4 lysozyme domain and one containing the receptor seven-helix bundle. One copy of each search model was found, producing a single solution. Subsequent refinement was performed using the MR solution with rounds of PHENIX.AutoBuild^53^ and PHENIX.Rosetta_Refine followed by PHENIX.Refine with simulated annealing, and Refmac5 using translation, libration and screw-rotation (TLS) parameters^54^. TLS displacement groups used in the refinement were defined by the TLSMD server^55^. Manual examination and rebuilding of refined coordinates was accomplished using COOT^56^. The NTSR1-EL-T4L structure was refined with final R/R_free_ values of 25.3/28.3. The quality of the model was checked using the Molprobity server^57^. The Ramachandran statistics for NTSR1-EL-T4L are: 98.5% favoured regions, 1.5% allowed regions, 0% outliers. The signal to noise ratio of I/σ(I) was ≥ 2.0 at 3.4 Å for NTSR1-EL-T4L. A summary of data collection and refinement statistics is reported in Supplementary Table 5. A stereo figure of representative electron density for TM7 of NTSR1-EL-T4L is given in Supplementary Fig. 3.

For NTSR1-EL-T4L, initial crystal screening was done at the Stanford Synchrotron Radiation Lightsource, beamline 12-2.

Figures were prepared in PyMOL (Schrödinger). Structural alignments were done with the ‘align’ command of PyMOL.

### Docking of SR48692 into the ligand binding pocket of NTSR1

The neurotensin agonist was removed from the crystal structure of NTSR1-LF (A86L^1.54^, E166A^3.49^, G215A^ECL2^, V360A^7.44^) (PDB code 4XES) (ref. 18), and the side chains of L86^1.54^, A166^3.49^, A215^ECL2^, and A360^7.44^ were changed to their respective wild-type amino acids (A86^1.54^, E166^3.49^, G215^ECL2^, V360^7.44^). The proposed SR48692 binding site^28^, based on mutagenesis data for orienting the antagonist, is partially occluded by the side chains of the residues Y146^3.29^, R149^3.32^, R328^6.55^ and Y324^6.51^ in the NTSR1-LF crystal structure. These residues were thus changed to alanine prior to docking the ligand.

The partial charges of the SR48692 ligand atoms were calculated based on electrostatic potential using *Jaguar* (Schrödinger Inc.). Using these charges, 60 distinct ligand conformations were generated outside the receptor using the program *Macromodel* (Schrödinger Inc.). These antagonist conformations were docked into NTSR1 using *GlideXP* (Schrödinger Inc.) imposing two experimentally derived constraints^28^; a salt bridge between R327^6.54^ and the carboxylic acid moiety of the antagonist, and the criteria that the dimethoxyphenyl moiety of SR48692 is within 4.5 Å of F358^7.42^. The docked conformations that satisfied these two distance constraints were further filtered using favorable contacts with the experimentally implicated residues M208^ECL2^, F331^6.58^, Y351^7.35^, F358^7.42^ and Y359^7.43^ (ref. 28). Finally, the side chains of A146^3.29^, A149^3.32^, A328^6.55^ and A324^6.51^ were mutated back to their wild type forms. Then the side chain rotamer conformations of all residues within 5 Å of SR48692 were optimized using *Prime* (Schrödinger Inc.), followed by minimization of the potential energy of the entire receptor-ligand complex using *Macromodel* (Schrödinger Inc.). The resulting model is called NTSR1-SR_dock_.

For comparison, the model NTSR1-NTS_dock_ was generated from the crystal structure of NTSR1-LF (PDB code 4XES) (ref. 18), with the side chains of L86^1.54^, A166^3.49^, A215^ECL2^, and A360^7.44^ changed to their respective wild-type amino acids (A86^1.54^, E166^3.49^, G215^ECL2^, V360^7.44^). The rotamer conformations of F358^7.42^ were generated using the program SCREAM (Side Chain Rotamer Energy Analysis Methodology)^58^.

### Molecular dynamics simulations

#### Preparation of the receptor structures

The molecular dynamics (MD) simulations for NTSR1-ELF_MD_ and NTSR1-EL_MD_ in the presence or absence of agonist were started from their respective crystal structures [PDB code 4XEE for NTSR1-ELF (ref. 18) and PDB code 5T04 for NTSR1-EL]. In all MD simulations, the T4 lysozyme of the crystallization constructs was omitted and the resulting carboxy and amino termini were capped at the end of TM5 and the start of TM6. The disordered residues in ICL1 (R91-Q99) of the NTSR1-EL crystal structure were homology modelled using Modeler v9.7 (refs 59 and 60). All MD simulations were performed in the presence of the peptide NTS_8-13_ or in the absence of ligand.

#### MD simulation protocols

MD simulations were performed with the GROMOS96 force field^61^ using the software package of GROMACS v4.6 (ref. 62) in 2 fs time steps. The NTSR1_MD_ systems were embedded in a hydrated palmitoyl-oleoyl-phosphatidyl-choline (POPC) bilayer, and all atoms including receptor, lipid, and waters were explicitly presented. For the packing of POPC molecules, we used the *inflateGRO* (ref. 63) packing package in GROMACS. All systems were first equilibrated using 200 ps of constant volume and temperature (NVT) ensemble at 310 K. Then, the systems were equilibrated in the constant pressure and temperature (NPT) ensemble using gradually reduced harmonic position restraints from 5 to 1 kcal/mol/Å^2^ applied to all heavy atoms of the protein and peptide ligand NTS_8-13_ during each of 5 ns. In the final NPT equilibration run, all positional restraints were released and run for 10 ns. Production simulations were initiated from the final snapshot of the equilibration run. After NVT equilibration followed by stepwise NPT equilibration, we performed five different production runs with different initial velocities, with each run up to 100 ns.

#### Analysis protocols

##### Calculation of energy contribution

The energy contributions were calculated from the non-bonded potential energy including Coulombic and Lennard-Jones terms over all MD ensemble trajectories. The components of all energy contributions consisted of the stability of the receptor and the interactions of protein/ligand, protein/POPC, and protein/waters.

##### Trajectory analysis

We analysed all 500 ns trajectories from MD simulations by GROMACS analysis tools and Python/Tcl scripting as needed. Visual Molecular Dynamics (VMD) and affiliated Tcl script were used for the visualization and structural conformation analysis of the MD trajectories. For the interhelical hydrogen bond (IHB) and hydrophobic (interhelical van der Waals, IvdW) interactions over the whole trajectories, the *g_hbond* and *g_contact* tools of GROMACS were used, respectively. To obtain a stable interaction of more than 50% of duration during 500 ns simulations, *contactFreq* of the Tcl script was used. To investigate the presence of water in the sodium-binding pocket of NTSR1_MD_, we used the *contacts_water* Tcl script for calculating the average number of water molecules within 3 Å of each chosen residue in the receptor.

##### Selection of representative structures

We first clustered the conformations in the MD trajectories by their root mean square deviation (RMSD) in coordinates of the main chain atoms in the transmembrane helices, using the *g_cluster* module in GROMACS with *gromos* clustering algorithm. An RMSD cut-off of 1.5 Å was used on the MD trajectories containing snapshots taken every 50 ps. Only main chain atoms of transmembrane helices were considered in the RMSD-clustering to reduce noise generated by the flexible loops. The representative structure was calculated as the frame that has the smallest RSMD to the center of the most populated conformational cluster.

## Additional Information

**Accession codes:** Coordinates and structure factors for NTSR1-EL-T4L are deposited in the Protein Data Bank under the accession code 5T04.

**How to cite this article**: Krumm, B. E. *et al*. Structure and dynamics of a constitutively active neurotensin receptor. *Sci. Rep.* **6**, 38564; doi: 10.1038/srep38564 (2016).

**Publisher's note:** Springer Nature remains neutral with regard to jurisdictional claims in published maps and institutional affiliations.

## Supplementary Material

Supplementary Information
