# Supplementary material for: Structure and dynamics of a constitutively active neurotensin receptor
Source: Sci Rep. 2016 Dec 7;6:38564. doi: 10.1038/srep38564 (PMC5141500; doi:10.1038/srep38564)
Supplement: Supplementary Information [file srep38564-s1.pdf]

**Supplementary Information**

**Structure and dynamics of a constitutively active neurotensin receptor**

Brian E. Krumm, Sangbae Lee, Supriyo Bhattacharya, Istvan Botos, Courtney F. White, Haijuan Du, Nagarajan Vaidehi, Reinhard Grisshammer

**Supplementary Figure 1 | Spare receptors.** HEK293 cells were transfected with varying amounts of the pcDNA3 plasmid derivative encoding NTSR1-WT. The IP production at 10 μM NTS was recorded using the IP-One HTRF kit. Data in the presence of NTS were normalized for each plasmid concentration to 100% of the respective value at t = 0 min, and are presented as the inverse of the normalized signal ratio of 665 nm / 620 nm. The NTSR1-WT expression levels were determined by [$^3$H]NTS binding (expressed as dpm values and receptor/cell numbers). Three independent experiments were conducted. The IP response appears saturated at a receptor density of ~ 2,000 dpm per well (**a**) corresponding to ~ 100,000 receptors/cell (**b**). Error bars correspond to s.e.m.

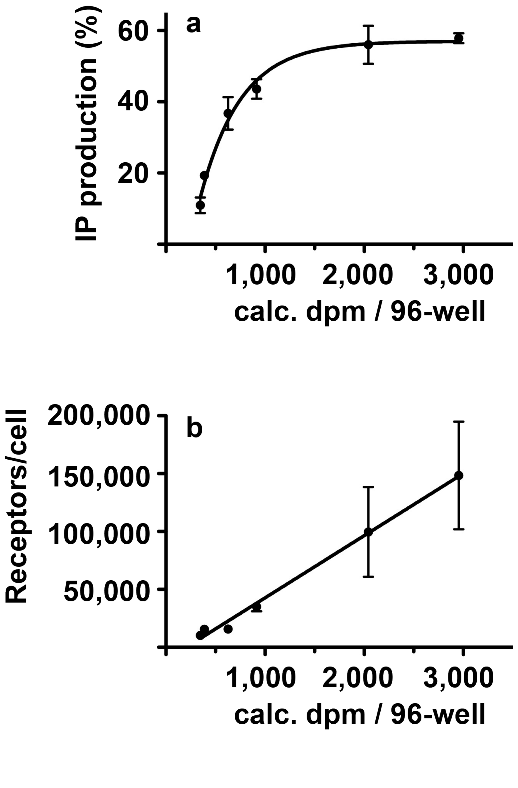

**Supplementary Figure 2 | Kinetic properties of NTSR1-EL.** Agonist association and dissociation. The binding experiments were conducted with neurotensin receptor constructs in urea-washed P2 insect cell membranes. The panel has the following colour code: NTSR1-WT (ref. 1) (orange); NTSR1-EL (red) with an ICL3 identical to that in NTSR1-WT; NTSR1-EL-T4L (blue) with most of ICL3 replaced with T4L. [$^3$H]NTS association (closed squares) was probed in TEBB buffer. Dissociation of agonist from NTSR1 was determined by quantifying the amount of [$^3$H]NTS remaining bound to receptors upon addition (arrow) of excess unlabelled NTS in the presence (open circles) or absence of NaCl (closed circles). Representative experiments conducted in singles are shown. Two independent experiments for each construct gave similar results.

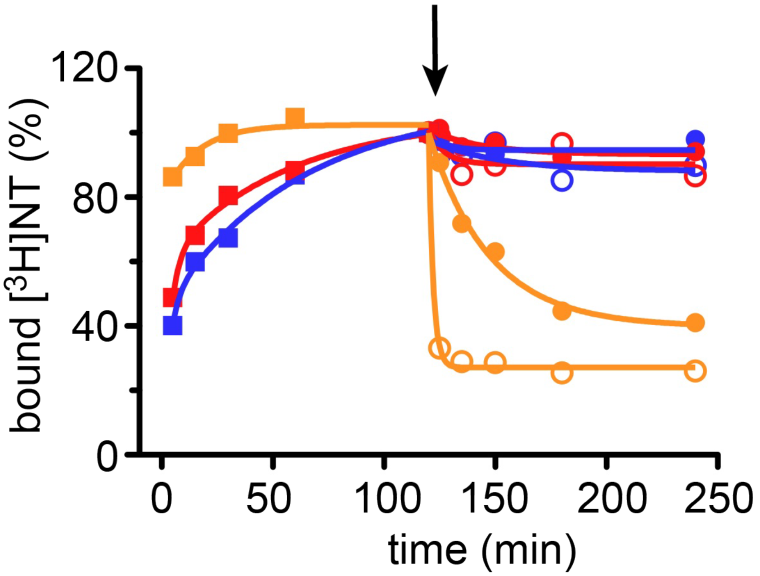

**Supplementary Figure 3 | Representative electron density showing TM7 of NTSR1-EL-T4L.** Stereoview of SigmaA weighted 2mFo-DFc maps contoured at 1σ. The map was generated using the SigmaA program of the CCP4 suite and the Phenix mtz2maps function.

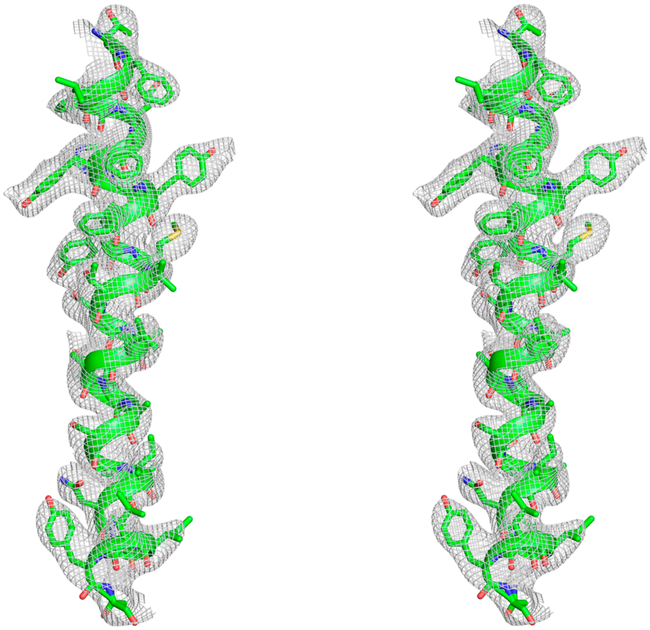

**Supplementary Figure 4 | The hydrophobic cascade.** NTSR1-EL-T4L (PDB code 5T04) (**a**) and NTSR1-ELF-T4L (PDB code 4XEE) (**b**) are shown in light-blue and green, respectively. Individual residues are shown as a stick model and are labelled. A series of stacking interactions relate Y324$^{6.51}$ to F358$^{7.42}$, W321$^{6.48}$ and F317$^{6.44}$ in NTSR1-ELF-T4L (ref. 2). This network is severed in NTSR1-EL-T4L owing to the F358A$^{7.42}$ stabilizing mutation.

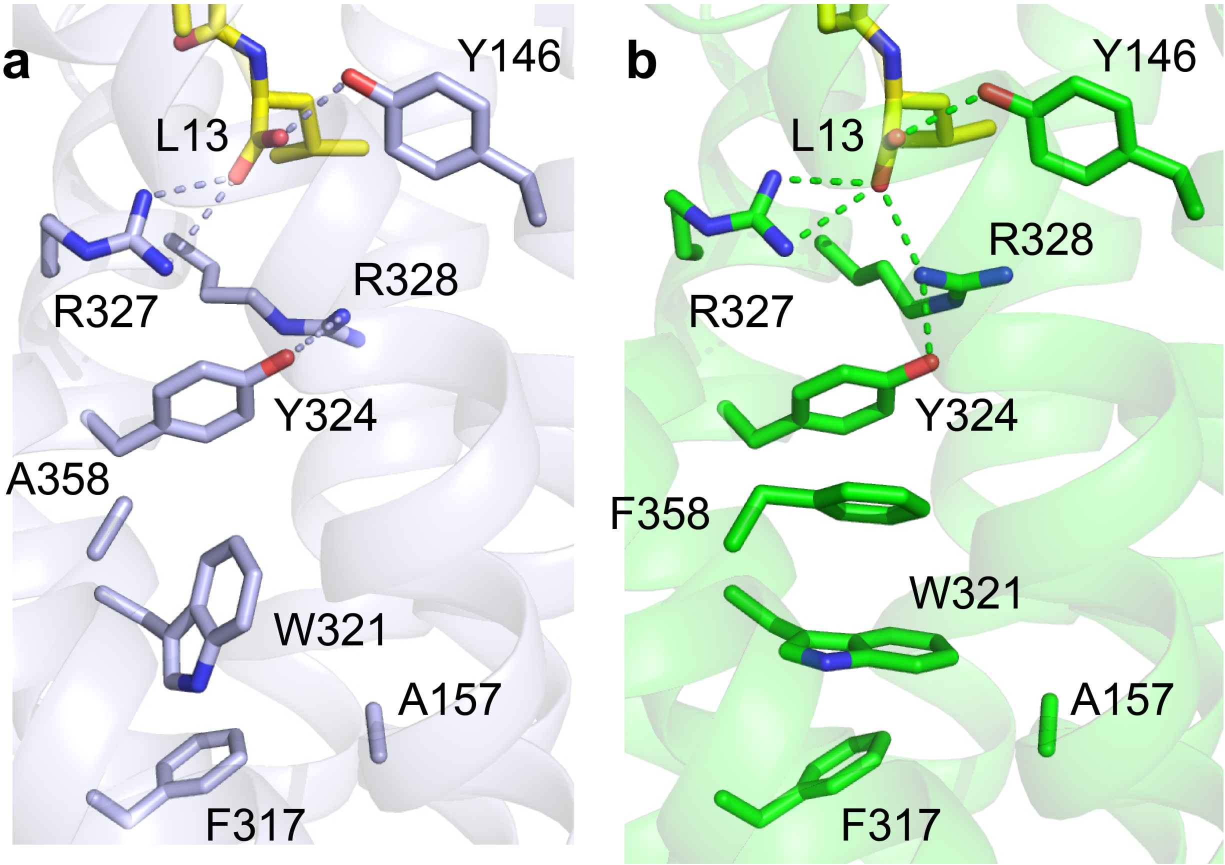

**Supplementary Figure 5 | The neurotensin binding pocket.** Cartoon representation of (**a**) NTSR1-EL-T4L (light blue) and (**b**) NTSR1-ELF-T4L (green) (PDB code 4XEE). $NTS_{8-13}$ is depicted as a stick model (orange, yellow). Dashed lines indicate hydrogen bonds. T4L has been omitted from the intracellular view for clarity. Individual residues are shown as a stick model and are labelled.

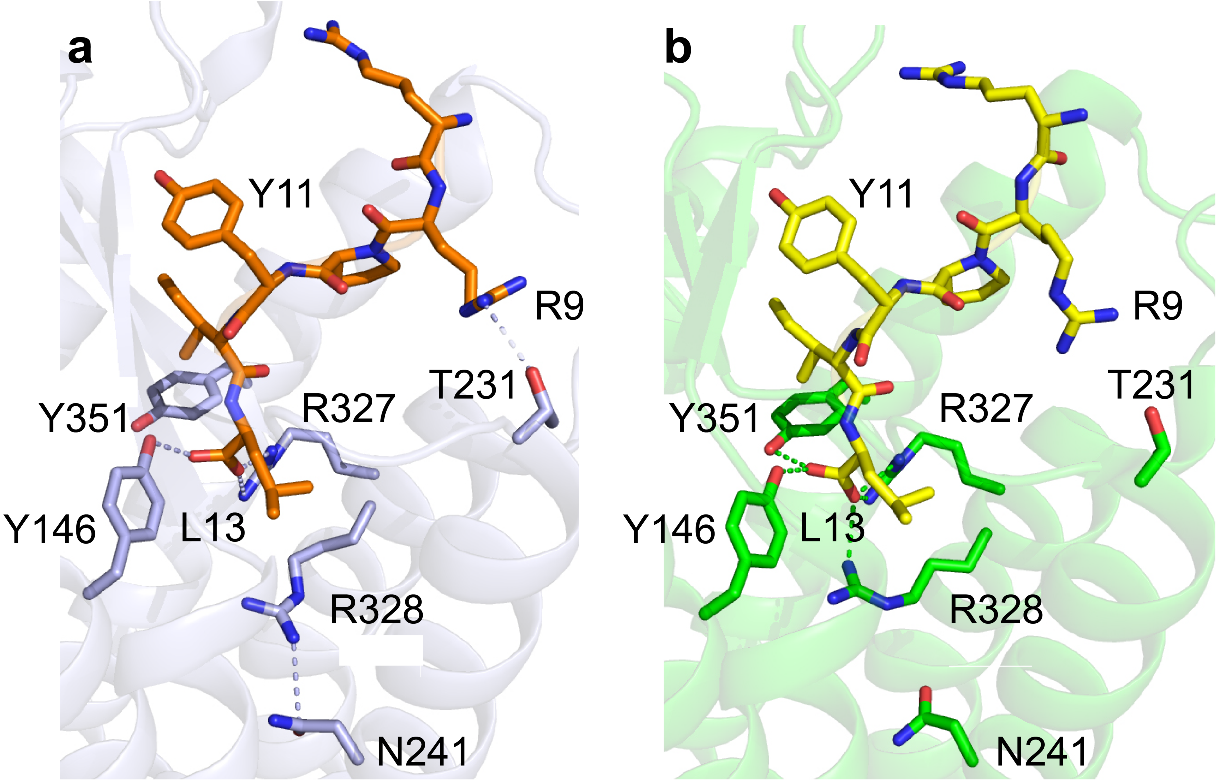

**Supplementary Figure 6 | The connector region.** Extracellular view of NTSR1-EL-T4L (light blue) and NTSR1-ELF-T4L (green) (PDB code 4XEE). $NTS_{8\text{-}13}$ and the top and bottom parts of NTSR1 have been omitted for clarity. Residues A157$^{3.40}$, V160$^{3.43}$, F246$^{5.47}$, P249$^{5.50}$ and F317$^{6.44}$ are depicted as a stick model and are labelled. W321$^{6.48}$ is shown as line model. Dashed lines indicate van der Waals interactions. Distances between Cα atoms and minimum inter-residue distances are given in Supplementary Tables 7 and 9. The arrow indicates the slight inward shift of TM3 in NTSR1-EL-T4L.

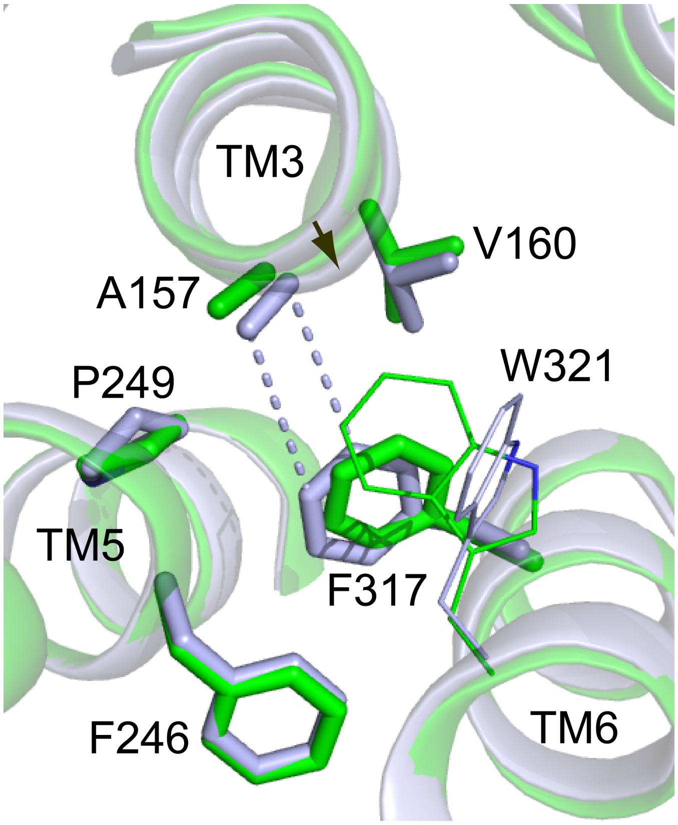

**Supplementary Figure 7 | Energetics of agonist-occupied NTSR1$_{MD}$.** (**a**) The calculated total non-bond energy of each system averaged over the entire dynamics trajectories is compared with the experimentally measured denaturation temperature ($T_m$) of agonist-occupied NTSR1-GW5, NTSR1-EL, NTSR1-ELF and NTSR1-WT (Supplementary Table 10). (**b**) The calculated non-bond energy of the respective NTSR1$_{MD}$ ensembles is the sum of the receptor non-bond energy, the interaction energy between receptor and the POPC bilayer, and receptor-water interactions. The most significant enthalpy contribution to the receptor stability comes from the protein internal energy and less from receptor-lipid and receptor-water interactions. Data for NTSR1-GW5$_{MD}$ are from ref. 3. Error bars correspond to s.d.

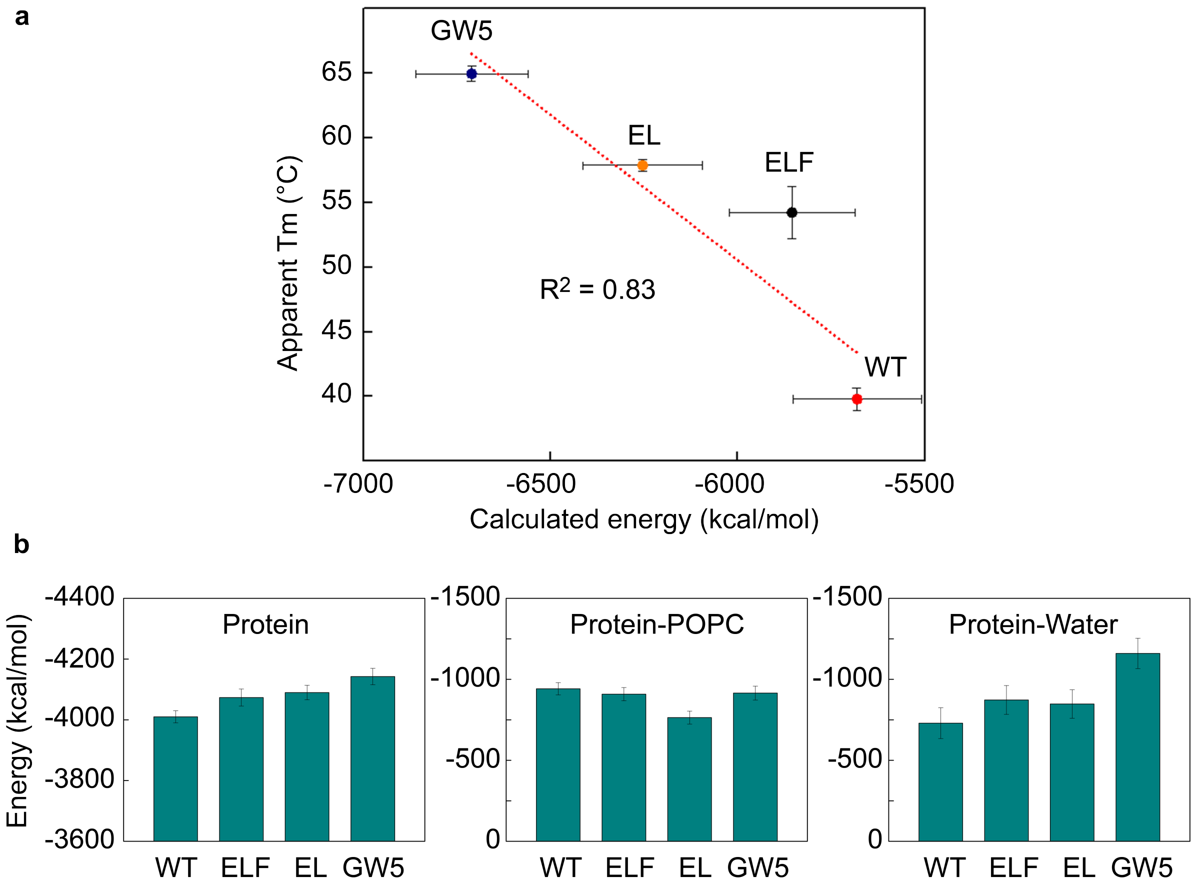

**Supplementary Figure 8 | The connector region of NTSR1-EL-T4L and TM86V-ΔIC3A.** (**a**) Side view in cartoon representation (NTSR1-EL-T4L in light blue, TM86V-ΔIC3A in light pink). Selected residues are shown as stick model. (**b**) Extracellular view. The N-terminus and ECLs have been omitted for clarity. Arrows indicate shifts.

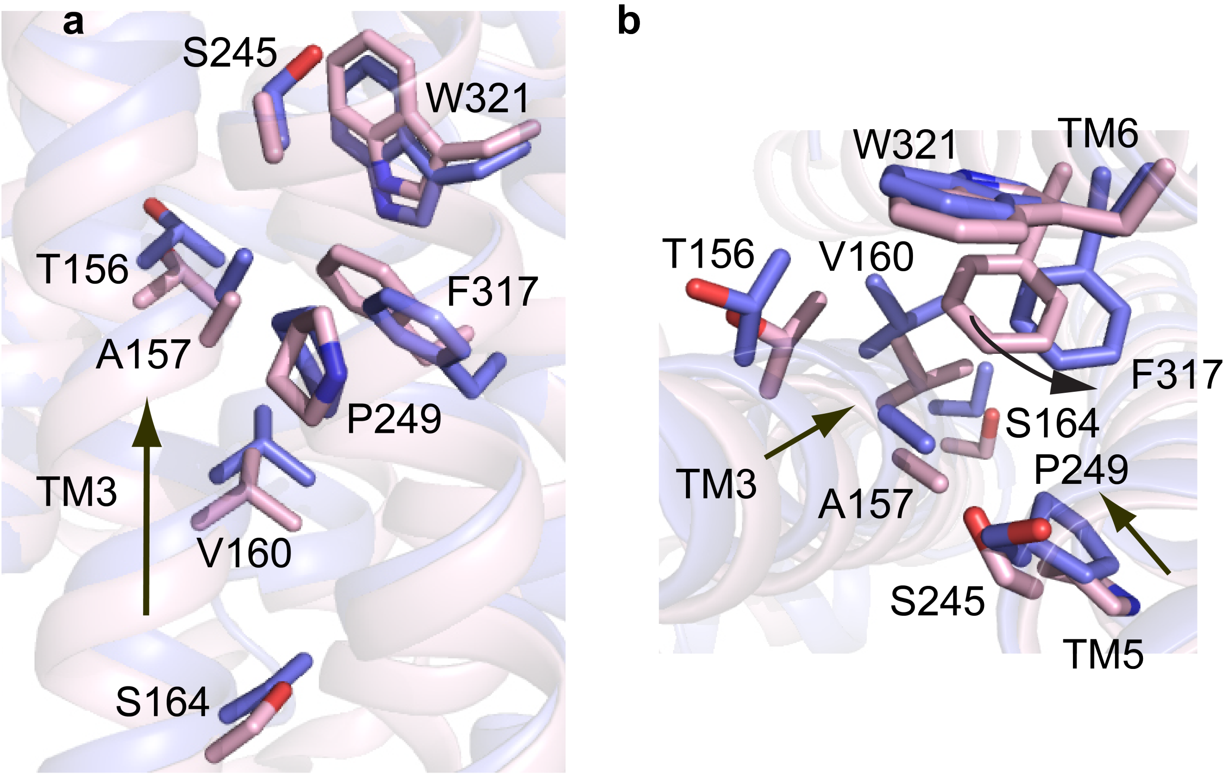

**Supplementary Table 1 | NTSR1 constructs used for pharmacological analyses.** Key features of the baculovirus constructs and pcDNA3/pME-HA derivatives are listed. All constructs used for GTPγS assays (urea-washed membranes from baculovirus infected insect cells) and inositol phosphate determination (transfected HEK293T or HEK293 cells) contained the wild-type ICL3 sequence. W321$^{6.48}$ of NTSR1 is a highly conserved residue within the CWxP motif. Superscripts are the Ballesteros-Weinstein numbers[4]; N-terminal tag, hemagglutinin signal peptide followed by the Flag tag; C-terminal tag, A-H10-GG; WT, wild-type.

| | | | |
|---|---|---|---|
| **Thermostabilized NTSR1 variants** | | | |
| | Mutations | | Reference |
| GW5 | A86L$^{1.54}$, E166A$^{3.49}$, G215A$^{ECL2}$, L310A$^{6.37}$, F358A$^{7.42}$, V360A$^{7.44}$ | | 1 |
| ELF | A86L$^{1.54}$, G215A$^{ECL2}$, V360A$^{7.44}$ | | 2 |
| EL | A86L$^{1.54}$, G215A$^{ECL2}$, F358A$^{7.42}$, V360A$^{7.44}$ | | 2, this study |
| TM86V | A86L$^{1.54}$, H103D$^{2.40}$, H105Y$^{2.42}$, A161V$^{3.44}$, R167L$^{3.50}$, R213L$^{ECL2}$, V232L$^{5.33}$, I253A$^{5.54}$, H305R$^{6.32}$, F358V$^{7.42}$, S362A$^{7.46}$ | | 5 |
| **Baculovirus constructs** (WT, ELF, EL) | | | |
| | N-terminal tag | Receptor residues | C-terminal tag |
| | Yes | T43-K396 | Yes |
| **pcDNA3 plasmids** (WT, W321A, ELF, ELF-W321A, EL, EL-W321A) | | | |
| | N-terminal tag | Receptor residues | C-terminal tag |
| | No | Met-H2-Y424 | No |
| **pME-HA** (TM86V) | No | Met-T43-K396 | No |

**Supplementary Table 2 | Signalling responses of NTSR1.** For inositol phosphate production, HEK293T cells were transfected with the respective NTSR1 constructs, or with the pcDNA3 plasmid as control. The effect of NTS on IP accumulation was recorded using the IP-One HTRF kit. $EC_{50\,(NTS)\,IP}$, half maximal effective concentration of NTS on IP accumulation. For GDP/[$^{35}$S]GTPγS exchange assays, binding experiments were conducted with NTSR1 constructs in urea-washed P2 insect cell membranes and purified Gq protein. $EC_{50\,(NTS)\,GTP\gamma S}$, half maximal effective concentration of NTS on the exchange of GDP for GTPγS on Gq. NTSR1-EL-T4L (n=1) did not stimulate nucleotide exchange in response to NTS. Data were analysed by nonlinear regression with the GraphPad Prism three-parameter dose-response equation, except when indicated otherwise. Fold-stimulation by NTS, fold-stimulation of the signalling response in the presence of saturating concentrations of NTS compared to the signalling response in the absence of ligand. A value of 1 indicates the absence of an NTS-catalysed signalling response. [a] Data from ref. 2. [b] Four parameter variable slope equation: The dose response curves for the determination of the half maximal effective concentration of NTS on the exchange of GDP for [$^{35}$S]GTPγS on Gq are multiphasic for NTSR1-ELF and wild-type receptor indicating high and low affinity agonist-binding sites at non-saturating G protein concentrations[2]. In contrast, the monophasic behaviour of NTSR1-EL indicates a single class of binding sites. [c] The means of the $EC_{50\,(NTS)\,IP}$ values for NTSR1-WT and NTSR1-Y324A are different (unpaired, two-tailed t test; P=0.01). As the expression level of NTSR1-Y324A is half of that of NTSR1-WT (Supplementary Table 3), a left-shift of the dose-response curve may occur if the expression level of NTSR1-Y324A would match that of NTSR1-WT. All values are given ± s.e.m. from independent experiments. n, number of independent experiments conducted; n.d., value not determined because addition of NTS did not result in an appreciable response above basal; no exp., no experiment conducted.

| | **WT** | **ELF** | **EL** | **Control** |
|---|---|---|---|---|
| $EC_{50\,(NTS)\,IP}$ (nM) | 7.0 ± 1.2 (n=26) | 1.5 ± 0.3 (n=7) | 4.1 ± 2.7 (n=4) | n.d. (n=4) |
| $EC_{50\,(NTS)\,GTP\gamma S}$ (nM) | 37 ± 13 (n=4)[a,b] | 4.7 ± 0.8 (n=3)[a,b] | 5.5 ± 0.6 (n=3) | no exp. |
| Fold-stimulation by NTS IP | 1.8 ± 0.1 (n=20) | 1.5 ± 0.1 (n=5) | 1.2 ± 0.1 (n=5) | 1.0 ± 0.0 (n=5) |
| Fold-stimulation by NTS GTPγS | 7.0 ± 0.6 (n=7)[a] | 5.6 ± 0.3 (n=10) | 3.3 ± 0.2 (n=6) | no exp. |

| | **W321A** | **ELF-W321A** | **EL-W321A** | **Y324A** |
|---|---|---|---|---|
| $EC_{50\,(NTS)\,IP}$ (nM) | 1.5 ± 0.2 (n=5) | 1.1 ± 0.3 (n=5) | 1.2 ± 0.2 (n=5) | 15 ± 3 (n=20)[c] |
| Fold-stimulation by NTS IP | 1.5 ± 0.1 (n=4) | 1.3 ± 0.1 (n=4) | 1.4 ± 0.0 (n=4) | 1.6 ± 0.1 (n=20) |

**Supplementary Table 3 | Expression levels of NTSR1 in transiently transfected HEK293T cells.** Specific surface receptors / cell; n, number of independent experiments conducted. [a] Average value of 830 and 860.

| Construct | Receptors/cell (x 1,000) | n | fold |
|---|---|---|---|
| WT | 86 ± 6 | 22 | 1 |
| ELF | 550 ± 45 | 12 | 6 |
| EL | 290 ± 35 | 12 | 3 |
| W321A | 230 ± 26 | 18 | 3 |
| ELF-W321A | 740 ± 66 | 18 | 9 |
| EL-W321A | 500 ± 42 | 16 | 6 |
| Y324A | 40 ± 6 | 7 | 0.5 |
| TM86V | 850[a] | 2 | 10 |

**Supplementary Table 4 | Ligand binding properties of NTSR1-EL constructs.** All binding experiments were conducted with NTSR1 constructs in urea-washed P2 insect cell membranes. NTSR1-EL-T4L with T4L replacing most of ICL3 was compared to NTSR1-EL containing ICL3. NTSR1-WT, NTSR1-ELF-T4L and NTSR1-ELF (ref. 2) were included as reference. All values are given ± s.e.m. from independent experiments conducted as single data points. All data were best fit to equations with a Hill slope of 1. $K_d$, equilibrium dissociation constant (saturation binding). Note that, in contrast to wild-type NTSR1, agonist binding to NTSR1-EL-T4L, NTSR1-EL, as well as to NTSR1-ELF-T4L and NTSR1-ELF did not reach equilibrium within the incubation time because of the slow [$^3$H]NTS off-rates. $IC_{50\ (SR48692)}$, half maximal inhibitory concentration of SR48692 on [$^3$H]NTS binding (heterologous competition). $K_{i\ (SR48692)}$, apparent dissociation constant for binding of inhibitor. The apparent $K_i$ values of NTSR1-EL-T4L and NTSR1-EL for the antagonist SR48692 (ref. 6) are 50-fold higher than the corresponding wild-type value. The large shift in the $K_i$ value may be caused by the F358A mutation, reducing the affinity of the antagonist[7] for NTSR1-EL and/or because SR48692 and [$^3$H]NTS binding to the NTSR1 mutants does not reach equilibrium under the experimental conditions because of the change in the off-rate of [$^3$H]NTS. $IC_{50\ (NaCl)}$, half maximal inhibitory concentration of $Na^+$ ions on [$^3$H]NTS binding. n, number of independent experiments; n.d., value not determined. [a] Data from ref. 2; [b] Value for apparent dissociation constant.

| | **WT**[a] | **ELF-T4L**[a] | **ELF**[a] | **EL-T4L** | **EL** |
|---|---|---|---|---|---|
| $K_d$ (nM) | 1.9 ± 0.3 (n=3) | 1.7 ± 0.1 (n=3)[b] | 1.3 ± 0.1 (n=4)[b] | 1.8 ± 0.1 (n=3)[b] | 2.2 ± 0.4 (n=6)[b] |
| $IC_{50\ (SR48692)}$ (nM) | 19 ± 7 (n=3) | 66 ± 5 (n=3) | 49 ± 10 (n=3) | 890 ± 90 (n=3) | 980 ± 80 (n=3) |
| $K_{i\ (SR48692)}$ (nM) | 5.2 ± 2.0 (n=3) | 17 ± 1 (n=3) | 10 ± 2 (n=3) | 230 ± 20 (n=3) | 300 ± 20 (n=3) |
| $IC_{50\ (NaCl)}$ (mM) | 56 ± 3 (n=3) | 360 ± 40 (n=3) | 430 ± 80 (n=3) | 520 ± 120 (n=3) | 270 ± 20 (n=3) |

**Supplementary Table 5 | Data collection and refinement statistics.**

| | **NTSR1-EL-T4L** |
|---|---|
| **Data Collection** | |
| Space group | $P\ 2_1 2_1 2$ |
| Mol/ASU | 1 |
| Cell dimensions | |
| *a, b, c* (Å) | 104.2, 75.7, 83.2 |
| α, β, γ (°) | 90, 90, 90 |
| Resolution (Å) | 41.6-3.3 (3.56-3.30)* |
| $R_{merge}$ (%) | 0.18 (0.72) |
| Mean I/σ(I) | 8.3 (1.6) |
| Completeness (%) | 97.3 (88.3) |
| Redundancy | 7.1 (3.7) |
| | |
| **Refinement** | |
| Resolution (Å) | 38.1-3.30 (3.41-3.30) |
| No. total reflections | 71001 (6681) |
| No. unique reflections | 10042 (1354) |
| $R_{work}/R_{free}$ (%) | 25.3/28.3 |
| No. atoms | |
| Protein | 3602 |
| Ligand | 101 |
| Water | 0 |
| *B*-factors (Å$^2$) | |
| NTSR1-T4L | 85.2 |
| NTSR1 | 81.7 |
| T4L | 91.5 |
| $NTS_{8-13}$ | 77.1 |
| R.m.s. deviations | |
| Bond lengths (Å) | 0.002 |
| Bond angles (°) | 0.494 |

Number of crystals for NTSR1-EL-T4L was 13.

* Highest resolution shell is shown in parenthesis

**Supplementary Table 6 | Hydrogen bonds and salt bridges between $NTS_{8\text{-}13}$ and NTSR1-EL-T4L.** The analysis was performed using the PDBePISA server[8]. Abbreviations and symbols used are as follows: H, hydrogen bond; S, salt bridge; TM, transmembrane helix; N, amino-terminus; ECL, extracellular loop.

| $NTS_{8\text{-}13}$ atoms | | Distance (Å) | NTSR1 atoms | | Location in NTSR1 |
|---|---|---|---|---|---|
| Arg8 | [N] | 3.9 (H) | Trp339 | [O] | ECL3 |
| Arg8 | [NH1] | 3.7 (H) | Ser53 | [O] | N |
| Arg8 | [NH1] | 3.7 (H) | Asp56 | [O] | N |
| Arg8 | [NH1] | 3.1 (H) | Asp54 | [O] | N |
| Arg9 | [NH1] | 2.8 (H,S) | Asp336 | [OD1] | ECL3 |
| Arg9 | [NH1] | 3.1 (S) | Asp336 | [OD2] | ECL3 |
| Arg9 | [NH2] | 3.1 (H) | Thr231 | [OG1] | TM5 |
| Arg9 | [NH2] | 3.8 (S) | Asp336 | [OD1] | ECL3 |
| Tyr11 | [OH] | 2.5 (H) | Leu55 | [O] | N |
| Tyr11 | [O] | 2.7 (H) | Thr226 | [OG1] | ECL2 |
| Ile12 | [O] | 2.9 (H) | Tyr347 | [OH] | TM7 |
| Leu13 | [N] | 3.5 (H) | Tyr146 | [OH] | TM3 |
| Leu13 | [O] | 2.7 (H,S) | Arg327 | [NH1] | TM6 |
| Leu13 | [O] | 3.3 (S) | Arg327 | [NH2] | TM6 |
| Leu13 | [OXT] | 2.7 (H) | Tyr146 | [OH] | TM3 |

**Supplementary Table 7 | Packing of TM3 against TM6 in NTSR1 crystal structures.** The distances between the Cα atoms of the indicated residues are listed for NTSR1-ELF-T4L (PDB code 4XEE), NTSR1-LF-T4L (PDB code 4XES), NTSR1-EL-T4L (PDB code 5T04), NTSR1-GW5-T4L (PDB code 4GRV) and TM86V-ΔIC3A (PDB code 3ZEV). The W321$^{6.48}$ and F317$^{6.44}$ side chain rotamer orientations are indicated by their respective dihedral $\chi_2$ or $\chi_1$ angles. =, parallel W321 side chain position; ⊥, upward W321 side chain position. All NTSR1 crystal structures have been determined in the presence of neurotensin.

| **Residues** | **ELF (4XEE)** | **LF (4XES)** | | **EL (5T04)** | **GW5 (4GRV)** | **TM86V (3ZEV)** |
|---|---|---|---|---|---|---|
| | **Cα-Cα distance (Å)** | | | | | |
| T153$^{3.36}$ - W321$^{6.48}$ | 10.3 | 10.5 | > | 9.8 | 9.8 | 9.4 |
| Y154$^{3.37}$ - W321$^{6.48}$ | 12.1 | 14.6 | ≥ | 11.5 | 11.6 | 12.1 |
| A157$^{3.40}$ - F317$^{6.44}$ | 9.3 | 9.4 | > | 8.4 | 8.7 | 8.2 |
| V160$^{3.43}$ - F317$^{6.44}$ | 9.2 | 9.5 | ≥ | 8.4 | 8.5 | 9.3 |
| **Rotamer orientation** | **χ angles** | | | | | |
| W321 ($\chi_2$) | 39° = | 34° = | | 103° ⊥ | 102° ⊥ | 98° ⊥ |
| F317 ($\chi_1$) | -163° | -163° | | -171° | 178° | -173° |

**Supplementary Table 8 | Packing of TM3 against TM6 in MD simulations.** The distances between the C$\alpha$ atoms of the indicated residues for NTSR1-ELF$_{MD}$ and NTSR1-EL$_{MD}$ are the average over the entire MD trajectories with standard deviations given in parenthesis. The predominating W321$^{6.48}$ and F317$^{6.44}$ side chain rotamer orientations are indicated by their respective dihedral $\chi_2$ or $\chi_1$ angles (peak values of distributions, Fig. 5). =, parallel W321 side chain position; ⊥, upward W321 side chain position, ↲, downward W321 side chain position. MD simulations have been performed in the presence (+NTS) or absence (-NTS) of agonist.

| | **+NTS** | | **-NTS** | **+NTS** | | **-NTS** |
|---|---|---|---|---|---|---|
| **Residues** | **ELF$_{MD}$** | | | **EL$_{MD}$** | | |
| | **C$\alpha$-C$\alpha$ distance (Å)** | | | | | |
| T153$^{3.36}$ - W321$^{6.48}$ | 9.9 (± 0.2) | ≥ | 9.8 (± 0.3) | 9.3 (± 0.2) | $<$ | 10.1 (± 0.2) |
| Y154$^{3.37}$ - W321$^{6.48}$ | 12.0 (± 0.2) | ≥ | 11.8 (± 0.2) | 11.7 (± 0.2) | ≤ | 11.9 (± 0.2) |
| A157$^{3.40}$ - F317$^{6.44}$ | 9.5 (± 0.3) | ≤ | 9.7 (± 0.2) | 9.8 (± 0.2) | ≥ | 9.5 (± 0.3) |
| V160$^{3.43}$ - F317$^{6.44}$ | 9.8 (± 0.4) | $<$ | 10.8 (± 0.2) | 10.5 (± 0.2) | ≥ | 10.2 (± 0.3) |
| **Rotamer orientation** | **$\chi$ angles** | | | | | |
| W321 ($\chi_2$) | 55° = | | -25° ↲ | 120° ⊥ | | 120° ⊥<br>60° = |
| F317 ($\chi_1$) | -170°<br>-80° | | -60° | -170° | | -170°<br>-90° |

**Supplementary Table 9 | Packing of the connector in receptor crystal structures.** Inter-residue distances between the indicated amino acids are calculated as the minimum distance between any side chain atoms (excluding hydrogen atoms) of the two residues listed. The PDB codes for the respective receptor structures are indicated. Distances for TM86V-ΔIC3A are shown for molecule A (ref. 5).

| **Side chain atom distance (Å)** | **ELF (4XEE)** | **EL (5T04)** | **TM86V-ΔIC3A (3ZEV)** | **GW5 (4GRV)** | **LF (4XES)** | |
|---|---|---|---|---|---|---|
| $P249^{5.50}$–$F317^{6.44}$ | 4.3 | 3.6 | 5.7 | 4.3 | 4.5 | |
| $A157^{3.40}$–$P249^{5.50}$ | 4.4 | 4.0 | 3.8 | 4.4 | 4.3 | |
| $A157^{3.40}$-$F317^{6.44}$ | 5.0 | 4.1 | 3.9 | 4.5 | 4.7 | |
| | **$\beta_2$AR active (3SN6)** | **$\beta_2$AR inactive (2RH1)** | **M2R active (4MQS)** | **M2R inactive (3UON)** | **MOR active (5C1M)** | **MOR inactive (4DKL)** |
| $P^{5.50}$–$F^{6.44}$ | 5.1 | 7.8 | 5.2 | 6.5 | 3.9 | 7.6 |
| $X^{3.40}$-$P^{5.50}$ | 3.8 | 3.7 | 4.0 | 5.3 | 3.4 | 3.6 |
| $X^{3.40}$-$F^{6.44}$ | 3.4 | 3.7 | 3.5 | 3.6 | 4.1 | 4.0 |

**Supplementary Table 10 | Denaturation temperature ($T_m$) of NTSR1-EL and NTSR1-EL-T4L in detergent solution.** NTSR1 constructs were expressed in insect cells. Denaturation profiles were recorded in the presence of [$^3$H]NTS and the detergent mixture LMNG-CHS. All values are given ± s.e.m. from 3 independent experiments conducted as single data points. Data for NTSR1-WT (Met-T43-Y424) and NTSR1-GW5-T4L are from ref. 1. Data for NTSR1-GW5, NTSR1-ELF-T4L and NTSR1-ELF are from ref. 2. [a,b] The means of the apparent $T_m$ values are significantly different (unpaired, two-tailed t test; P<0.05).

| | **Apparent $T_m$ (ºC)** | **$\Delta T_m$ (ºC)** |
|---|---|---|
| NTSR1-WT | 40 ± 1 | |
| NTSR1-ELF-T4L | 51 ± 1 | 11 |
| NTSR1-ELF | 54 ± 1 | 14 |
| NTSR1-EL-T4L[a] | 54 ± 0 | 14 |
| NTSR1-EL[a] | 58 ± 0 | 18 |
| NTSR1-GW5-T4L[b] | 59 ± 0 | 19 |
| NTSR1-GW5[b] | 65 ± 0 | 25 |

**Supplementary Table 11 | Residues stabilizing the active receptor state.** Side chain minimum inter-residue distances for $Y^{5.58}$ and $Y^{7.53}$ of active GPCRs are compared to those of NTSR1. The respective PDB codes are given in parenthesis.

| Receptor | Residues | Distance (Å) |
|---|---|---|
| Active $\beta_2$AR (3SN6) | Y219-Y326 | 4.3 |
| Active μOR (5C1M) | Y252-Y336 | 4.6 |
| Active M2R (4MQS) | Y206-Y440 | 4.2 |
| NTSR1-ELF (4XEE) | N257-Y369 | 5.8 |
| NTSR1-EL (5T04) | N257-Y369 | 6.9 |
| TM86V-ΔIC3A (3ZEV) | N257-Y369 | 12.2 |

**Supplementary Table 12 | Interhelical contacts of NTSR1$_{MD}$.** Numbers represent the interhelical hydrogen bond and van der Waals interactions that are present in more than 50% of the conformations in the MD trajectories. BB, backbone; IC, intracellular receptor side; IHB, interhelical hydrogen bond; IvdW, interhelical van der Waals contact; SC, side chain; TM, transmembrane domain; #, number of contacts. The highlighted contacts are shown in Fig. 7.

| | -NTS | +NTS | -NTS | +NTS |
|---|---|---|---|---|
| | ELF$_{MD}$ | | EL$_{MD}$ | |
| All TMs | | | | |
| IHB | 33 | 34 | 35 | 40 |
| IvdW | 55 | 54 | 51 | 57 |
| Total | 88 | 88 | 86 | 97 |
| IC | | | | |
| IHB | 14 | 11 | 12 | 15 |
| IvdW | 28 | 24 | 26 | 28 |
| Total | 42 | 35 | 38 | 43 |

IHB

| | -NTS | | | | +NTS | | | | -NTS | | | | +NTS | | | |
|---|---|---|---|---|---|---|---|---|---|---|---|---|---|---|---|---|
| | ELF$_{MD}$ | | | | | | | | EL$_{MD}$ | | | | | | | |
| TM1-TM2 | | | | | | | | | | | | | N82 | SC | D113 | BB |
| | | | | | | | | | | | | | N82 | SC | L114 | BB |
| TM1-TM7 | N82 | SC | S362 | BB | N82 | SC | S362 | BB | N82 | SC | S362 | BB | N82 | SC | S362 | BB |
| TM2-TM3 | T101 | SC | E166 | SC | T101 | SC | E166 | SC | T101 | SC | E166 | SC | T101 | SC | E166 | SC |
| | | | | | | | | | H105 | SC | N159 | SC | | | | |
| | H105 | SC | S162 | SC | H105 | SC | S162 | SC | H105 | SC | S162 | SC | H105 | SC | S162 | SC |
| | S108 | SC | N159 | SC | S108 | SC | N159 | SC | S108 | SC | N159 | SC | S108 | SC | N159 | SC |
| | S112 | SC | T156 | SC | S112 | SC | T156 | SC | | | | | S112 | SC | T156 | SC |
| | D113 | SC | T156 | SC | | | | | | | | | D113 | SC | T156 | SC |
| | S100 | SC | T186 | SC | | | | | | | | | | | | |
| TM2-TM4 | S108 | SC | W194 | SC | S108 | SC | W194 | SC | S108 | SC | W194 | SC | S108 | SC | W194 | SC |
| TM2-TM7 | D113 | SC | S362 | SC | D113 | SC | S362 | SC | D113 | SC | S362 | SC | D113 | SC | S362 | SC |
| | | | | | | | | | | | | | D113 | SC | N365 | SC |
| | E124 | SC | Y359 | SC | E124 | SC | Y359 | SC | E124 | SC | Y359 | SC | E124 | SC | Y359 | SC |
| TM3-TM4 | | | | | | | | | E166 | SC | R185 | SC | E166 | SC | R185 | SC |

| | | | | | | | | |
|---|---|---|---|---|---|---|---|---|
| TM3-TM5 | S164 SC | N257 SC | S164 SC | N257 SC | S164 SC | N257 SC | | |
| TM3-TM7 | R149 SC<br>S164 SC | Y359 SC<br>Y369 SC | R149 SC | Y359 SC | R149 SC | Y359 SC | R149 SC | Y359 SC |
| TM5-TM6 | N257 SC | L310 BB | N257 SC | L310 BB | N257 SC | L310 BB | N257 SC | L310 BB |
| # | 14 | | 11 | | 12 | | 15 | |

IvdW

| | -NTS | | +NTS | | -NTS | | +NTS | |
|---|---|---|---|---|---|---|---|---|
| | $ELF_{MD}$ | | | | $EL_{MD}$ | | | |
| TM1-TM2 | A86 | A110 | A86 | A110 | L86 | A110 | A86 | A110 |
| | L89 | L106 | L89 | L106 | L89 | L106 | L89 | L106 |
| TM2-TM3 | | | H105 | L163 | | | | |
| | L109 | T156 | | | L109 | T156 | L109 | T156 |
| | L109 | V160 | | | L109 | V160 | L109 | V160 |
| | L109 | L163 | L109 | L163 | | | | |
| TM2-TM4 | T101 | T186 | T101 | T186 | T101 | T186 | T101 | T186 |
| | Y104 | T186 | Y104 | T186 | Y104 | T186 | Y104 | T186 |
| | | | Y104 | I190 | Y104 | I190 | Y104 | I190 |
| TM2-TM7 | | | | | L106 | Y369 | L106 | Y369 |
| | | | | | | | L109 | Y369 |
| | | | I116 | Y359 | | | | |
| TM3-TM4 | L158 | I193 | L158 | I193 | L158 | I193 | L158 | I193 |
| TM3-TM5 | V160 | I253 | V160 | I253 | V160 | I253 | V160 | I253 |
| | V165 | L256 | V165 | L256 | V165 | L256 | V165 | L256 |
| | Y168 | L256 | Y168 | L256 | Y168 | L256 | Y168 | L256 |
| | Y168 | V259 | Y168 | V259 | Y168 | V259 | Y168 | V259 |
| | Y168 | I260 | Y168 | I260 | Y168 | I260 | Y168 | I260 |
| | I171 | I260 | I171 | I260 | | | I171 | I260 |
| TM3-TM6 | V160 | F317 | | | | | | |
| TM3-TM7 | V160 | Y369 | V160 | Y369 | V160 | Y369 | V160 | Y369 |
| | L163 | Y369 | L163 | Y369 | L163 | Y369 | L163 | Y369 |
| TM5- | M250 | F317 | M250 | F317 | | | M250 | F317 |

| | | | | | | | | |
|---|---|---|---|---|---|---|---|---|
| TM6 | M250 | V318 | | | M250 | V318 | | |
| | I253 | V314 | I253 | V314 | | | I253 | V314 |
| | | | | | | | I253 | F317 |
| | I260 | L310 | I260 | L310 | I260 | L310 | I260 | L310 |
| | A261 | V307 | A261 | V307 | A261 | V307 | A261 | V307 |
| | L264 | L303 | L264 | L303 | L264 | L303 | L264 | L303 |
| | L264 | V307 | | | T265 | V307 | | |
| TM6-TM7 | V309 | L368 | | | V309 | L368 | V309 | L368 |
| | | | | | V309 | L371 | | |
| | V309 | V372 | V309 | V372 | | | V309 | V372 |
| | V313 | L368 | | | V313 | L368 | V313 | L368 |
| | V313 | Y369 | V313 | Y369 | V313 | Y369 | V313 | Y369 |
| | | | | | V313 | L371 | | |
| # | 28 | | 24 | | 26 | | 28 | |

**Supplementary references**


1 White, J. F. *et al*. Structure of the agonist-bound neurotensin receptor. *Nature* **490**, 508-513 (2012).

2 Krumm, B. E., White, J. F., Shah, P. & Grisshammer, R. Structural prerequisites for G-protein activation by the neurotensin receptor. *Nat. Commun.* **6**, 7895 (2015).

3 Lee, S., Bhattacharya, S., Tate, C. G., Grisshammer, R. & Vaidehi, N. Structural dynamics and thermostabilization of neurotensin receptor 1. *J. Phys. Chem. B* **119**, 4917-4928 (2015).

4 Ballesteros, J. A. & Weinstein, H. Integrated methods for the construction of three-dimensional models and computationla probing of structure-function relations in G protein-coupled receptors. *Methods Neurosci.* **25**, 366-428 (1995).

5 Egloff, P. *et al*. Structure of signalling-competent neurotensin receptor 1 obtained by directed evolution in Escherichia coli. *Proc. Natl. Acad. Sci. U.S.A.* **111**, E655-662 (2014).

6 Gully, D. *et al*. Biochemical and pharmacological profile of a potent and selective nonpeptide antagonist of the neurotensin receptor. *Proc. Natl. Acad. Sci. U.S.A.* **90**, 65-69 (1993).

7 Labbe-Jullie, C. *et al*. Mutagenesis and modeling of the neurotensin receptor NTR1. Identification of residues that are critical for binding SR 48692, a nonpeptide neurotensin antagonist. *J. Biol. Chem.* **273**, 16351-16357 (1998).

8 Krissinel, E. & Henrick, K. Inference of macromolecular assemblies from crystalline state. *J. Mol. Biol.* **372**, 774-797 (2007).
